# Capsular serovars of virulent *Capnocytophaga canimorsus* are shared by the closely related species *C. canis* and *C. cynodegmi*

**DOI:** 10.1038/s41426-018-0126-x

**Published:** 2018-07-04

**Authors:** Francesco Renzi, Estelle Hess, Melanie Dol, Dunia Koudad, Elodie Carlier, Maria Ohlén, Edward Moore, Guy Richard Cornelis

**Affiliations:** 1 Research Unit in Biology of Microorganisms (URBM), University of Namur, 5000 Namur, Belgium; 2 Culture Collection University of Gothenburg (CCUG), Department of Clinical Microbiology, Sahlgrenska University Hospital, 41346 Gothenburg, Sweden; 3 Department of Infectious Diseases, Sahlgrenska Academy, University of Gothenburg, 41346 Gothenburg, Sweden

## Abstract

*Capnocytophaga canimorsus* is a dog oral commensal bacterium that causes rare but life-threatening generalized infections in humans who have been in contact with its animal hosts. Two other dog commensals, *Capnocytophaga canis* and *Capnocytophaga cynodegmi*, cause rare, mild local infections. To date, nine capsular serovars have been described in *C. canimorsus*. Here, we serotyped 112 strains of *Capnocytophaga* spp. isolated from human infections. The *C. canimorsus* strains (86 of 96, 89.6%) belonged to serovars A, B, or C with relative frequencies of approximately 30% for each serovar. The high prevalence of the A, B, and C serovars in strains isolated from humans, compared to the previously described low prevalence of these serovars among dog isolates (7.6%), confirms that these three serovars are more virulent to humans than other serovars and suggests that the low incidence of disease may be linked to the low prevalence of the A, B, and C serovars in dogs. We serotyped six strains of *C. canis* and ten strains of *C. cynodegmi* and, surprisingly, found one *C. canis* and three *C. cynodegmi* strains to be of capsular serovar B. This observation prompted us to test 34 dog-isolated *C. canis* and 16 dog-isolated *C. cynodegmi* strains. We found four *C. canis* strains belonging to serovar A and one belonging to serovar F. In contrast, no dog-isolated *C. cynodegmi* strain could be typed with the available antisera. This work demonstrates that virulence-associated capsular polysaccharides (A, B, and C) are not specific to the *C. canimorsus* species.

## Introduction

*Capnocytophaga* is a Gram-negative bacterial genus in the phylum Bacteroidetes that represents part of the normal oral flora of domestic animals and humans^1^. Four *Capnocytophaga* species are present in dog and cat oral cavities: *C. canimorsus*; *C. cynodegmi*; *C. canis*; and *C. stomatis*^2–4^. *C. cynodegmi*, *C. canis*, and *C. stomatis* are associated with human mild wound infections, although, recently, one case of *C. canis*-mediated sepsis was described^5^. In contrast, *C. canimorsus* has been known since 1961 to cause severe generalized infections in patients who have been bitten, scratched, or simply in contact with dogs and cats^6–8^. Despite the administration of adequate antibiotherapy, *C. canimorsus-*induced septicemia may evolve to septic shock with mortality as high as 30% and debilitating morbidity in survivors^6^.

Several predisposing factors for infection have been identified in *C. canimorsus*-infected patients, such as splenectomy, alcohol abuse, smoking and advanced age, although as many as 40% of patients did not present any obvious risk factor^9^, implying that *C. canimorsus* cannot solely be considered an opportunistic pathogen. To date, more than 500 cases of human infections have been reported. Two studies have reported an incidence of 0.5–0.7 cases per million inhabitants per year^10,11^, although, in 2016, a retrospective study performed in the Helsinki area in Finland reported an incidence of 4.1 cases per million inhabitants per year^12^. The discrepancy between these estimates might result from the difficulty in diagnosing *C. canimorsus* infections, presumably because of the slow and fastidious growth of these bacteria in culture^2^.

Recently, we reported the discovery of a capsular polysaccharide at the surface of *C. canimorsus* cells, composed of the same polysaccharide units as the lipooligosaccharide O-antigen^13^. While the repertoire of capsular serovars is extensive among strains isolated from dog mouths, three serovars, named A, B, and C, are predominant, worldwide, among human infections^14^. Here, to further investigate the correlation between capsular serovars and virulence, we serotyped the *Capnocytophaga* strains obtained from the Culture Collection University of Gothenburg (CCUG), the largest collection of strains of *Capnocytophaga* spp. isolated from human infections. Our study confirms the high prevalence of capsular serovars A, B, and C among the strains of *C. canimorsus* isolated from human infections (86/96, 89.6%), and we describe two new serovars (L and M). The CCUG collection contains six human-isolated *C. canis* and nine *C. cynodegmi* strains. One *C. canis* strain turned out to belong to serovar B, as did three *C. cynodegmi* strains. This unexpected finding prompted us to test our collection of 34 *C. canis* strains isolated from dog mouths, and we found that four strains belonged to serovar A and one to serovar F.

Thus, our study confirms the higher virulence of capsular serovars A, B, and C of *C. canimorsus*, but also shows that serovars A, B, and F, and presumably others, described in *C. canimorsus* can be found in *C. canis* and *C. cynodegmi*.

## Results

### Capsular typing of human-isolated *C. canimorsus* strains

**Fig. 1:**
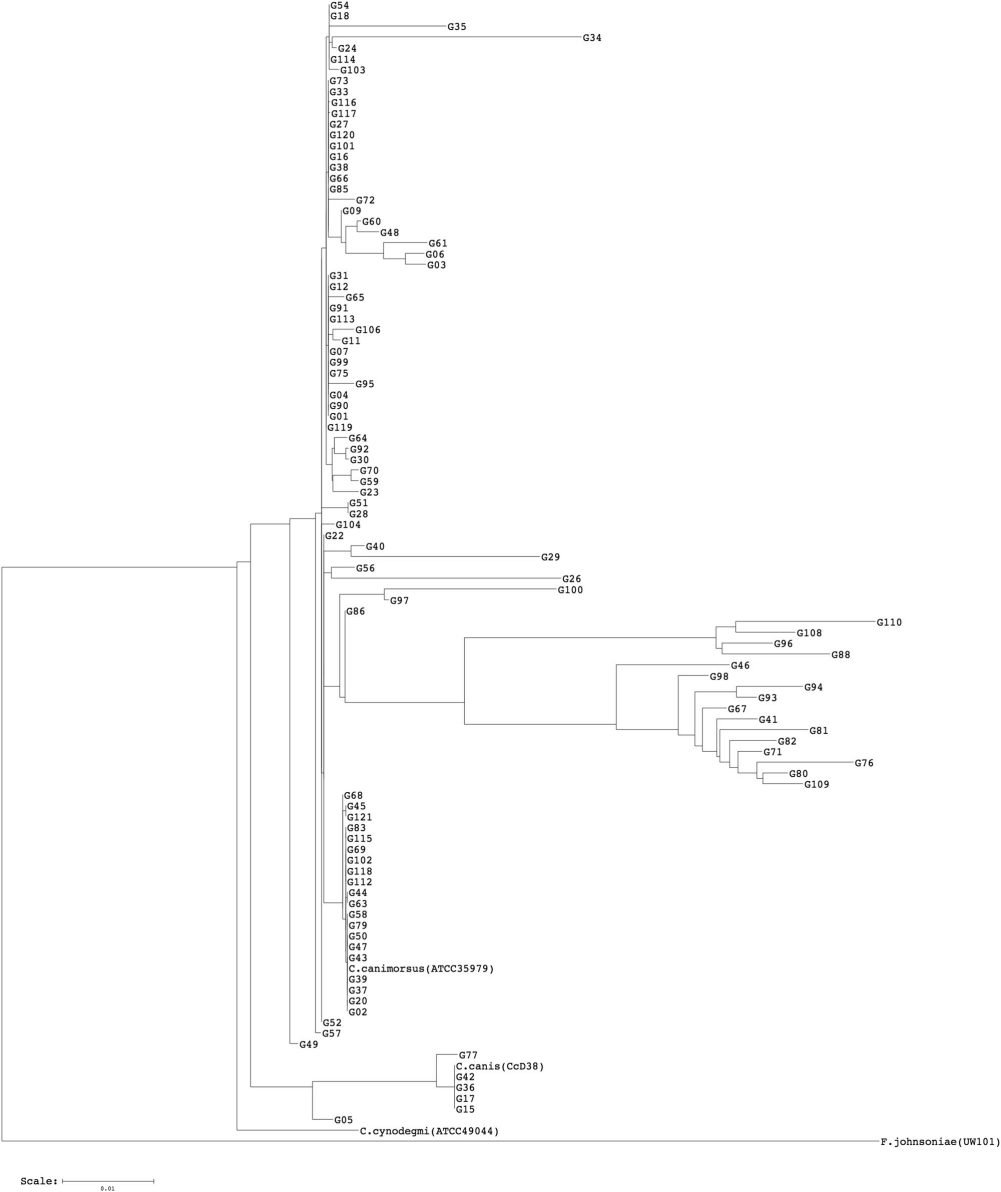
16S rDNA majority consensus tree of CCUG clinical strains. *Flavobacterium johnsoniae* (accession number M59051) was selected as an outgroup (software: RDP Tree Builder)

**Fig. 2:**
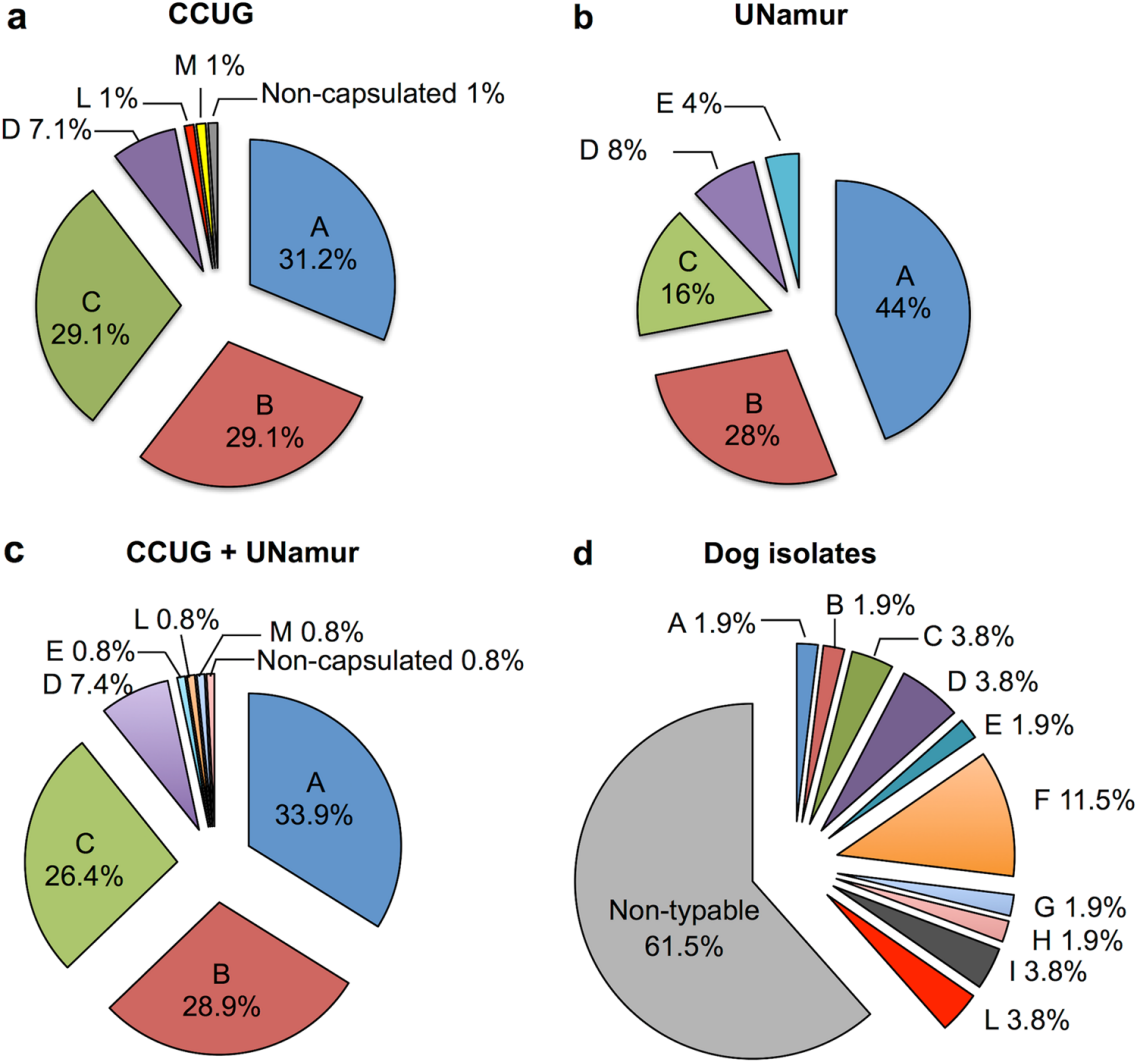
Prevalence of capsular serovars in *C. canimorsus* strains. Capsular serovar prevalence in human-isolated *C. canimorsus* strains from the CCUG collection (**a**), UNamur collection (**b**), and CCUG and UNamur collections (**c**). Capsular serovar prevalence in dog-isolated *C. canimorsus* strains (**d**). The data presented in **b** and **d** were partially previously published^14^

Since the *C. canis* species has been only recently described^3–5^, we tested whether any of the 102 strains from the CCUG identified as *C. canimorsus* might be reclassified as *C. canis*. To this end, we sequenced the PCR-amplified 16S rRNA genes (16S rDNAs) of these 102 strains and generated a dendrogram of their estimated phylogenetic relationships (Fig. 1). The dendrogram clearly showed that 96 strains (94.1%) clustered with the *C. canimorsus* type strain (ATCC 35979), while six strains (5.9%) clustered with the *C. canis* type strain (CcD38 = LMG 29146) (Fig. 1). According to the 16S rDNA phylogenetic analysis, these six strains were, thus, reclassified as *C. canis* (Supplementary Table S1). We then subjected the 96 *C. canimorsus sensu stricto* strains to the PCR test designed to detect the three A, B, and C capsular serovars^14^. These analyses showed that 87 of the 96 strains were positive and could be further typed as either A, B, or C by serovar-specific PCRs (Table 1 and Supplementary Fig. S1a–d). To confirm these results, we performed Western blot analyses on polysaccharide extracts of the 87 strains, using antisera specifically recognizing the A, B, or C capsular serovars (Table 1 and Supplementary Fig. S2a–c). This experiment confirmed the PCR typing results and identifications for all strains tested except for strain G58, which was found to belong to the B serovar by PCR but did not react with the B antiserum. In summary, 86 of the 96 *C. canimorsus* strains tested (89.58%) belonged to either serovars A (30/96, 31.3%), B (28/96, 29.2%), or C (28/96, 29.2%). Next, we tested all strains by PCRs detecting the D and E capsular types^14^. According to these analyses, seven strains belonged to serovar D and none to serovar E (Table 1 and Supplementary Fig. S1e, f), which was confirmed by Western blot (Supplementary Fig. S2d). We then tested the three strains that we could not type by the A-E PCRs (G06, G45, and G58) using Western blot for all the capsular types (A–I) we identified in previous studies^14^. None of the strains reacted with these antisera (Supplementary Fig. S2a–i). Three rabbit antisera were thus raised against the G06, G45, and G58 whole bacteria and adsorbed (see Methods). The anti-G06 antiserum strongly recognized the capsule of the G06 strain (Supplementary Fig. S2j). The anti-G58 serum recognized only the capsule of G58 (Supplementary Fig. S2k). Surprisingly, a Western blot analysis of a polysaccharide sample of strain G45 with G45 antiserum did not show any capsule for this strain (Supplementary Fig. S2l). Thus, we identified two new serovars that we named L (G06) and M (G58), and we found a strain without a capsule. The capsular typing results are summarized in Fig. 2. In short, we found that the 96 *C. canimorsus* strains from the CCUG belonged to only six serovars, with A, B, and C being heavily dominant (89.6%), followed by the D serovar (7.3%) (Fig. 2a). These results are in line with those previously observed for a collection of 25 strains isolated worldwide from human infections (Fig. 2a, b)^14^, with the only difference being that none of the CCUG strains belonged to the E serovar and one strain was non-capsulated.

**Table 1:** Capsular typing of 96 human-isolated *C. canimorsus* strains from the CCUG collection

Strain	PCR typing							WB typing									
	ABC	A	B	C	D	E	Serovar	A	B	C	D	E	F	G	H	I	Serovar
G01	+	+	+	−	−	−	A	+	nd	nd	nd	nd	nd	nd	nd	nd	A
G02	+	+	−	+	−	−	A	+	nd	nd	nd	nd	nd	nd	nd	nd	A
G03	+	−	+	−	−	−	B	nd	+	nd	nd	nd	nd	nd	nd	nd	B
G04	+	+	+	−	−	−	A	+	nd	nd	nd	nd	nd	nd	nd	nd	A
G06	−	−	−	−	−	−	nt	−	−	−	−	−	−	−	−	−	nt
G07	+	−	+	−	−	−	B	nd	+	nd	nd	nd	nd	nd	nd	nd	B
G09	+	−	+	−	−	−	B	nd	+	nd	nd	nd	nd	nd	nd	nd	B
G11	+	+	+	−	−	−	A	+	nd	nd	nd	nd	nd	nd	nd	nd	A
G12	+	−	+	−	−	−	B	nd	+	nd	nd	nd	nd	nd	nd	nd	B
G16	+	−	+	−	−	−	B	nd	+	nd	nd	nd	nd	nd	nd	nd	B
G18	+	−	−	+	−	−	C	nd	nd	+	nd	nd	nd	nd	nd	nd	C
G20	+	−	−	+	−	−	C	nd	nd	+	nd	nd	nd	nd	nd	nd	C
G22	+	−	−	+	−	−	C	nd	nd	+	nd	nd	nd	nd	nd	nd	C
G23	+	−	−	+	−	−	C	nd	nd	+	nd	nd	nd	nd	nd	nd	C
G24	+	+	+	−	−	−	A	+	nd	nd	nd	nd	nd	nd	nd	nd	A
G26	+	−	−	+	−	−	C	nd	nd	+	nd	nd	nd	nd	nd	nd	C
G27	−	−	−	−	+	−	D	nd	nd	nd	+	nd	nd	nd	nd	nd	D
G28	+	−	+	−	−	−	B	nd	+	nd	nd	nd	nd	nd	nd	nd	B
G29	+	−	−	+	−	−	C	nd	nd	+	nd	nd	nd	nd	nd	nd	C
G30	+	−	+	−	−	−	B	nd	+	nd	nd	nd	nd	nd	nd	nd	B
G31	+	−	+	−	−	−	B	nd	+	nd	nd	nd	nd	nd	nd	nd	B
G33	+	−	+	−	−	−	B	nd	+	nd	nd	nd	nd	nd	nd	nd	B
G34	+	+	+	−	−	−	A	+	nd	nd	nd	nd	nd	nd	nd	nd	A
G35	+	+	+	−	−	−	A	+	nd	nd	nd	nd	nd	nd	nd	nd	A
G37	+	−	−	+	−	−	C	nd	nd	+	nd	nd	nd	nd	nd	nd	C
G38	+	+	−	+	−	−	A	+	nd	nd	nd	nd	nd	nd	nd	nd	A
G39	+	−	−	+	−	−	C	nd	nd	+	nd	nd	nd	nd	nd	nd	C
G40	+	+	−	+	−	−	A	+	nd	nd	nd	nd	nd	nd	nd	nd	A
G41	+	+	+	−	−	−	A	+	nd	nd	nd	nd	nd	nd	nd	nd	A
G43	+	−	−	+	−	−	C	nd	nd	+	nd	nd	nd	nd	nd	nd	C
G44	+	−	+	−	−	−	B	nd	+	nd	nd	nd	nd	nd	nd	nd	B
G45	−	−	−	−	−	−	nt	−	−	−	−	−	−	−	−	−	nt
G46	+	−	−	+	−	−	C	nd	nd	+	nd	nd	nd	nd	nd	nd	C
G47	+	+	+	−	−	−	A	+	nd	nd	nd	nd	nd	nd	nd	nd	A
G48	+	−	+	−	−	−	B	nd	+	nd	nd	nd	nd	nd	nd	nd	B
G49	+	−	+	−	−	−	B	nd	+	nd	nd	nd	nd	nd	nd	nd	B
G50	+	−	−	+	−	−	C	nd	nd	nd	nd	nd	nd	nd	nd	nd	C
G51	+	−	+	−	−	−	B	nd	+	+	nd	nd	nd	nd	nd	nd	B
G52	+	−	+	−	−	−	B	nd	+	nd	nd	nd	nd	nd	nd	nd	B
G54	+	+	−	+	−	−	A	+	nd	nd	nd	nd	nd	nd	nd	nd	A
G56	+	−	−	+	−	−	C	nd	nd	+	nd	nd	nd	nd	nd	nd	C
G57	+	−	+	−	−	−	B	nd	+	nd	nd	nd	nd	nd	nd	nd	B
G58	+	−	+	−	−	−	B	−	−	−	−	−	−	−	−	−	nt
G59	+	+	+	−	−	−	A	+	nd	nd	nd	nd	nd	nd	nd	nd	A
G60	+	−	+	−	−	−	B	nd	+	nd	nd	nd	nd	nd	nd	nd	B
G61	+	−	+	−	−	−	B	nd	+	nd	nd	nd	nd	nd	nd	nd	B
G63	+	−	+	−	−	−	B	nd	+	nd	nd	nd	nd	nd	nd	nd	B
G64	+	+	+	−	−	−	A	+	nd	nd	nd	nd	nd	nd	nd	nd	A
G65	+	−	+	−	−	−	B	nd	+	nd	nd	nd	nd	nd	nd	nd	B
G66	−	−	−	−	+	−	D	nd	nd	nd	+	nd	nd	nd	nd	nd	D
G67	+	+	−	+	−	−	A	+	nd	nd	nd	nd	nd	nd	nd	nd	A
G68	+	+	−	+	−	−	A	+	nd	nd	nd	nd	nd	nd	nd	nd	A
G69	+	−	−	+	−	−	C	nd	nd	+	nd	nd	nd	nd	nd	nd	C
G70	+	−	−	+	−	−	C	nd	nd	+	nd	nd	nd	nd	nd	nd	C
G71	+	−	−	+	−	−	C	nd	nd	+	nd	nd	nd	nd	nd	nd	C
G72	+	−	+	−	−	−	B	nd	+	nd	nd	nd	nd	nd	nd	nd	B
G73	+	+	+	−	−	−	A	+	nd	nd	nd	nd	nd	nd	nd	nd	A
G75	+	−	+	−	−	−	B	nd	+	nd	nd	nd	nd	nd	nd	nd	B
G76	+	+	−	+	−	−	A	+	nd	nd	nd	nd	nd	nd	nd	nd	A
G79	+	−	−	+	−	−	C	nd	nd	+	nd	nd	nd	nd	nd	nd	C
G80	+	−	−	+	−	−	C	nd	nd	+	nd	nd	nd	nd	nd	nd	C
G81	+	−	−	+	−	−	C	nd	nd	+	nd	nd	nd	nd	nd	nd	C
G82	+	−	−	+	−	−	C	nd	nd	+	nd	nd	nd	nd	nd	nd	C
G83	+	+	+	−	−	−	A	+	nd	nd	nd	nd	nd	nd	nd	nd	A
G85	+	+	+	−	−	−	A	+	nd	nd	nd	nd	nd	nd	nd	nd	A
G86	−	−	−	−	+	−	D	nd	nd	nd	+	nd	nd	nd	nd	nd	D
G88	+	−	−	+	−	−	C	nd	nd	+	nd	nd	nd	nd	nd	nd	C
G90	−	−	−	−	+	−	D	nd	nd	nd	+	nd	nd	nd	nd	nd	D
G91	+	+	+	−	−	−	A	+	nd	nd	nd	nd	nd	nd	nd	nd	A
G92	+	+	+	−	−	−	A	+	nd	nd	nd	nd	nd	nd	nd	nd	A
G93	+	+	+	−	−	−	A	+	nd	nd	nd	nd	nd	nd	nd	nd	A
G94	+	−	−	+	−	−	C	nd	nd	+	nd	nd	nd	nd	nd	nd	C
G95	+	−	+	−	−	−	B	nd	+	nd	nd	nd	nd	nd	nd	nd	B
G96	+	−	−	+	−	−	C	nd	nd	+	nd	nd	nd	nd	nd	nd	C
G97	+	−	+	−	−	−	B	nd	+	nd	nd	nd	nd	nd	nd	nd	B
G98	+	+	+	−	−	−	A	+	nd	nd	nd	nd	nd	nd	nd	nd	A
G99	−	−	−	−	+	−	D	nd	nd	nd	+	nd	nd	nd	nd	nd	D
G100	+	−	−	+	−	−	C	nd	nd	+	nd	nd	nd	nd	nd	nd	C
G101	+	+	+	−	−	−	A	+	nd	nd	nd	nd	nd	nd	nd	nd	A
G102	+	−	+	−	−	−	B	nd	+	nd	nd	nd	nd	nd	nd	nd	B
G103	+	−	−	+	−	−	C	nd	nd	+	nd	nd	nd	nd	nd	nd	C
G104	+	−	+	−	−	−	B	nd	+	nd	nd	nd	nd	nd	nd	nd	B
G106	−	−	−	−	+	−	D	nd	nd	nd	+	nd	nd	nd	nd	nd	D
G108	+	+	−	+	−	−	A	+	nd	nd	nd	nd	nd	nd	nd	nd	A
G109	+	+	+	−	−	−	A	+	nd	nd	nd	nd	nd	nd	nd	nd	A
G110	+	−	−	+	−	−	C	nd	nd	+	nd	nd	nd	nd	nd	nd	C
G112	+	+	+	−	−	−	A	+	nd	nd	nd	nd	nd	nd	nd	nd	A
G113	+	−	+	−	−	−	B	nd	+	nd	nd	nd	nd	nd	nd	nd	B
G114	+	+	+	−	−	−	A	+	nd	nd	nd	nd	nd	nd	nd	nd	A
G115	+	−	+	−	−	−	B	nd	+	nd	nd	nd	nd	nd	nd	nd	B
G116	+	+	+	−	−	−	A	+	nd	nd	nd	nd	nd	nd	nd	nd	A
G117	+	−	−	+	−	−	C	nd	nd	+	nd	nd	nd	nd	nd	nd	C
G118	+	−	−	+	−	−	C	nd	nd	+	nd	nd	nd	nd	nd	nd	C
G119	+	−	−	+	−	−	C	nd	nd	+	nd	nd	nd	nd	nd	nd	C
G120	+	−	+	−	−	−	B	nd	+	nd	nd	nd	nd	nd	nd	nd	B
G121	−	−	−	−	+	−	D	nd	nd	nd	+	nd	nd	nd	nd	nd	D

Capsular typing was determined by PCR using the oligonucleotides given in Supplementary Table S2. The results were interpreted as previously described^14^: strains positive for PCR A, PCR A and PCR B or PCR A and PCR C were typed as A, strains positive for only PCR B were typed as B, and strains positive for only PCR C as C. Western blot analysis on polysaccharidic structures was performed using specific polyclonal rabbit antisera. *nd* not determined, *nt* non-typable

### Screening of dog-isolated *C. canimorsus* strains for the presence of the L and M capsular serovars

We tested the prevalence of the new L and M capsular serovars in a collection of 52 *C. canimorsus* strains isolated from the mouths of dogs from Switzerland and Belgium^3,14^ by an ELISA. Two strains, one isolated in Switzerland and one isolated in Belgium, were found to react with the anti-L sera (CcD20 and CcD106), while none reacted with the anti-M sera (Supplementary Table S3). These results were confirmed by Western blot analysis of polysaccharidic structures (Supplementary Fig. S3). This experiment showed that capsular serovar L is not limited to Scandinavia.

### Capsular typing of *C. canis* strains isolated from human infections reveals the presence of a serovar B strain

As mentioned above, the strains of *Capnocytophaga* spp. in this study that were isolated from human clinical samples included six *C. canis* strains (Fig. 1 and Supplementary Table S1). This finding may seem odd because *C. canis* was described initially as a non-pathogenic species^3^, although, since then, a few cases of human infections from *C. canis* have been reported. Zangenah et al. reported the isolation of a *C. canis* from a wound caused by a cat bite, and Taki et al. recently described a human septicemia after a cat bite^4,5^. It was, thus, not completely unexpected to find some *C. canis* among the collection of clinical strains, although the limited number of *C. canis* strains (6) compared to the high number of *C. canimorsus* (96) strains in the strain collection indicates that *C. canis* is far less involved in human infections than *C. canimorsus*.

The majority of *C. canis* strains isolated from dogs lack several factors that might contribute to *C. canimorsus* pathogenicity, such as the ability to acquire iron from transferrin and proliferate in human serum, as well as the capacity to deglycosylate host glycoproteins and cytochrome-oxidase activity^3^. However, Zangenah et al. and Taki et al. reported that the *C. canis* isolates that caused human infections are endowed with cytochrome-oxidase activity, thus suggesting that these strains differ from the majority of *C. canis* strains found in dog mouths. We tested for oxidase activity in the six *C. canis* strains isolated from human clinical samples and observed two of them (G05 and G17) to be positive (data not shown).

Given the close relationship between *C. canimorsus* and *C. canis*, we assessed whether other factors, such as capsular serovars, might be shared between these two species and, in particular, among the clinical *C. canis* strains; we, thus, tested the six strains of *C. canis* isolated from human clinical samples for the presence of *C. canimorsus* capsular serovars by PCR, using the primers for serovars ABC, D, and E. Three strains out of six gave a positive result for the ABC PCR (G05, G36, and G77), but two of them (G36 and G77) could not be typed further by PCR using the A-specific, B-specific, and C-specific primers or by Western blot using the specific antisera. G05 was identified as serovar B (Table 2 and Supplementary Fig. S4a–f), and this result was confirmed by a Western blot analysis (Table 2 and Supplementary Fig. S5a). The remaining three *C. canis* strains were tested by Western blot for the presence of capsular serovars, but none of them reacted with any of the sera (Supplementary Fig. S5b–l). The finding that one of the *C. canis* human clinical strains (G05) has a *C. canimorsus* serovar B capsule is of great interest, since it shows for the first time that capsular serovar B is shared between these two closely related species. Interestingly, strain G05 is also oxidase-positive, thus suggesting that it might be more virulent than most *C. canis* strains, since it possesses these two factors. It is also of interest that one strain (G36) was isolated from a patient who had been bitten by a cat. This is the only cat-transmitted strain we have in our collections of human isolates.

**Table 2:** Capsular typing of six human-isolated *C. canis* strains from the CCUG collection

Strain	PCR typing							WB typing											
	ABC	A	B	C	D	E	Serovar	A	B	C	D	E	F	G	H	I	L	M	Serovar
G05	+	−	+	−	−	−	B	nd	+	nd	nd	nd	nd	nd	nd	nd	nd	nd	B
G15	−	−	−	−	−	−	nt	−	−	−	−	−	−	−	−	−	−	−	nt
G17	−	−	−	−	−	−	nt	−	−	−	−	−	−	−	−	−	−	−	nt
G36	+	−	−	−	−	−	nt	−	−	−	−	−	−	−	−	−	−	−	nt
G42	−	−	−	−	−	−	nt	−	−	−	−	−	−	−	−	−	−	−	nt
G77	+	−	−	−	−	−	nt	−	−	−	−	−	−	−	−	−	−	−	nt

Capsular typing was determined by PCR using the oligonucleotides given in Supplementary Table S2. The results were interpreted as indicated in ref. ^14^: strains positive for PCR A, PCR A and PCR B or PCR A and PCR C were typed as A, strains positive for only PCR B were typed as B, and strains positive for only PCR C as C. Western blot analysis on polysaccharidic structures was performed using specific polyclonal rabbit antisera. *nd* not determined, *nt* non-typable

### *C. canis* isolated from dogs includes serovar A strains

We also assessed whether other *C. canimorsus* capsular serovars might be shared by *C. canis*; we screened by PCR and ELISA our *C. canis* collection of 34 strains isolated from healthy dogs^3^. Four isolates (CcD7, CcD11, CcD46, and CcD111) were identified as capsular serovar A, and one (CcD123) was serovar F (Supplementary Fig. S6 and Table 3). None of the other strains of *C. canis* reacted with any of the tested sera (Table 3). We confirmed the presence of the capsular serovars A and F by Western blot analyses (Supplementary Fig. S7). Our data, thus, showed that, in addition to the serovar B capsule, *C. canis* can also be endowed with capsules of serovar A (four strains of 34) and of serovar F (one strain of 34). The four serovar A strains are oxidase-negative, while the serovar F strain is oxidase-positive^3^.

**Table 3:** Capsular typing of 34 dog-isolated *C. canis* strains

Strain CcD	PCR							ELISA												WB		
	ABC	A	B	C	D	E	Serovar	A	B	C	D	E	F	G	H	I	L	M	Serovar	A	F	Serovar
1	−	−	−	−	−	−	nt	7.3	6.5	12.4	14.7	11.8	14.6	15.4	9.5	12.9	9.6	8.3	nt	nd	nd	nt
4	−	−	−	−	−	−	nt	6.1	6.0	12.8	16.1	9.26	14.7	15.4	9.9	11.8	10.3	7.9	nt	nd	nd	nt
7	+	+	−	−	−	−	A	**102.6**	8.6	10.8	13.8	9.76	13.2	15.1	10.6	10.4	9.1	7.3	A	+	nd	A
11	+	+	−	−	−	−	A	**99.0**	6.9	9.98	14.4	9.35	12.8	14.9	10.3	11.1	8.8	7.3	A	+	nd	A
15	−	−	−	−	−	−	nt	9.6	6.9	10.2	12.9	8.85	12.7	14.5	9.8	10.2	9.9	7.8	nt	nd	nd	nt
36	−	−	−	−	−	−	nt	12.3	6.0	11.8	13.5	9.35	13.5	14.3	9.3	10.6	9.5	8.0	nt	nd	nd	nt
38	−	−	−	−	−	−	nt	8.7	6.2	11.5	14.3	9.93	14.6	14.5	10.1	11.1	11.1	9.2	nt	nd	nd	nt
46	+	+	−	−	−	−	A	**96.7**	6.4	11.3	13.5	9.10	12.2	14.0	8.7	9.8	11.6	8.5	A	+	nd	A
50	−	−	−	−	−	−	nt	8.7	6.7	10.9	12.1	8.77	11.9	13.4	8.7	8.8	9.0	7.3	nt	nd	nd	nt
54	−	−	−	−	−	−	nt	9.3	5.8	8.09	15.3	9.14	14.5	16.3	12.8	10.8	7.9	5.7	nt	nd	nd	nt
64	−	−	−	−	−	−	nt	9.0	6.6	11.0	14.1	10.1	14.3	15.0	10.1	10.7	8.7	6.9	nt	nd	nd	nt
66	−	−	−	−	−	−	nt	11.7	6.7	11.2	13.9	10.3	13.7	14.8	9.5	11.0	8.8	7.6	nt	nd	nd	nt
75	−	−	−	−	−	−	nt	9.2	6.9	10.7	12.9	9.76	12.5	15.7	9.3	9.6	9.4	7.9	nt	nd	nd	nt
79	−	−	−	−	−	−	nt	8.9	6.0	10.3	13.8	9.39	15.2	15.6	9.6	10.1	9.5	7.8	nt	nd	nd	nt
82	−	−	−	−	−	−	nt	9.1	6.8	10.0	15.2	11.3	13.1	15.1	9.8	11.0	9.1	7.2	nt	nd	nd	nt
85	−	−	−	−	−	−	nt	11.6	6.2	9.5	13.2	10.2	14.1	14.9	9.7	11.1	8.5	6.9	nt	nd	nd	nt
88	−	−	−	−	−	−	nt	10.3	6.2	10.6	12.8	10.2	13.1	14.3	8.8	10.3	10.2	7.6	nt	nd	nd	nt
93	−	−	−	−	−	−	nt	6.7	5.7	12.7	14.7	9.8	13.3	14.8	9.4	10.6	9.8	7.6	nt	nd	nd	nt
94	−	−	−	−	−	−	nt	8.2	6.76	8.63	12.81	9.31	12.6	13.7	9.6	10.2	6.8	5.8	nt	nd	nd	nt
95	−	−	−	−	−	−	nt	7.1	6.3	10.6	14.1	9.14	12.8	15.0	9.9	10.1	19.8	7.6	nt	nd	nd	nt
97	−	−	−	−	−	−	nt	6.7	6.6	10.7	13.5	9.02	13.8	15.7	9.8	9.8	9.9	8.2	nt	nd	nd	nt
102	−	−	−	−	−	−	nt	10.5	6.0	9.44	14.9	9.31	14.1	16.8	11.0	10.4	8.7	6.1	nt	nd	nd	nt
103	−	−	−	−	−	−	nt	7.7	9.6	8.57	15.3	10.8	14.4	16.5	10.8	10.9	9.5	7.0	nt	nd	nd	nt
108	−	−	−	−	−	−	nt	9.6	7.6	9.20	14.55	9.51	12.3	13.8	9.1	8.8	7.8	7.2	nt	nd	nd	nt
109	−	−	−	−	−	−	nt	7.9	6.5	10.1	13.0	10.14	12.5	15.1	8.7	9.2	10.0	7.4	nt	nd	nd	nt
110	−	−	−	−	−	−	nt	8.0	5.7	10.5	17	9.89	13.6	14.7	9.4	9.9	7.8	6.7	nt	nd	nd	nt
111	+	+	−	−	−	−	A	**104.5**	9.4	10.4	14.7	10.6	13.4	16.3	10.4	11.3	9.5	7.0	A	+	nd	A
112	−	−	−	−	−	−	nt	7.1	6.5	9.32	15.5	9.47	12.1	14.1	9.3	10.3	8.8	6.7	nt	nd	nd	nt
114	−	−	−	−	−	−	nt	8.9	6.8	11.4	15	9.85	13.4	16.2	9.8	9.9	11.3	7.7	nt	nd	nd	nt
121	−	−	−	−	−	−	nt	8.1	6.3	11.4	15.1	9.51	11.6	14.9	9.6	10.7	9.5	7.5	nt	nd	nd	nt
123	−	−	−	−	−	−	nt	10.3	9.2	7.3	21.9	9.16	**81.2**	13.7	12.0	10.7	21.4	10.3	F	nd	+	F
125	−	−	−	−	−	−	nt	7.4	7.8	11.1	13.8	7.29	12.5	15.4	13.3	9.0	8.2	6.5	nt	nd	nd	nt
127	−	−	−	−	−	−	nt	8.1	7.1	12.5	18.9	7.87	12.1	14.2	12.4	9.9	8.8	7.9	nt	nd	nd	nt
128	−	−	−	−	−	−	nt	7.4	16.8	12.3	13.	6.9	11.2	13.4	10.8	10.6	9.1	7.9	nt	nd	nd	nt

Capsular typing was determined by PCR using the oligonucleotides given in Supplementary Table S2. The results were interpreted as indicated in ref. ^14^: strains positive for PCR A, PCR A and PCR B or PCR A and PCR C were typed as A, strains positive for only PCR B were typed as B, and strains positive for only PCR C as C. Capsular serotyping was determined by ELISA on entire heat-killed bacteria using specific polyclonal rabbit antisera. The readout of the ELISA was absorbance, but the results are expressed here as percentage of reactivity calculated with respect to the absorbance value obtained for the capsular type strain. Values are expressed as the means. Isolates with high reactivities are in bold. Western blot analysis on polysaccharidic structures was performed using specific polyclonal rabbit antisera. *nd* not determined, *nt* non-typable

### Capsular serovar B is also found in *C. cynodegmi* human isolates

The finding that *C. canis* strains may have capsules of the same serovars that identify more virulent *C. canimorsus* strains raised the question of whether *C. cynodegmi*, another closely related species, might also have capsules of these *C. canimorsus* serovars. We tested 10 *C. cynodegmi* strains isolated from human infections and 16 *C. cynodegmi* strains isolated from healthy dogs (Supplementary Figs. S8, S9, S10) by PCR and Western blot. As summarized in Table 4, three of the 10 *C. cynodegmi* strains isolated from human infections were found to possess serovar B capsules (Supplementary Fig. S9). The remaining 7 human-isolated strains and the 16 dog-isolated strains could not be typed with the 11 *C. canimorsus* antisera (Table 4, Supplementary Figs. S9, S10). In conclusion, our data show that *C. cynodegmi*, as well as *C. canis*, may share capsule serovar B.

**Table 4:** Capsular typing of *C. cynodegmi* isolates

	Strain	PCR typing							WB typing											
		ABC	A	B	C	D	E	Serovar	A	B	C	D	E	F	G	H	I	L	M	Serovar
Human strains	G14	−	−	−	−	−	−	nt	−	−	−	−	−	−	−	−	−	−	−	nt
	G25	−	−	−	−	−	−	nt	−	−	−	−	−	−	−	−	−	−	−	nt
	G55	+	−	+	−	−	−	B	−	+	−	−	−	−	−	−	−	−	−	B
	G78	+	−	+	−	−	−	B	−	+	−	−	−	−	−	−	−	−	−	B
	G84	−	−	−	−	−	−	nt	−	−	−	−	−	−	−	−	−	−	−	nt
	G89	−	−	−	−	−	−	nt	−	−	−	−	−	−	−	−	−	−	−	nt
	G107	+	−	+	−	−	−	B	−	+	−	−	−	−	−	−	−	−	−	B
	G122	−	−	−	−	−	−	nt	−	−	−	−	−	−	−	−	−	−	−	nt
	G123	−	−	−	−	−	−	nt	−	−	−	−	−	−	−	−	−	−	−	nt
	Ccy4	−	−	−	−	−	−	nt	−	−	−	−	−	−	−	−	−	−	−	nt
Dog strains	Gd02	−	−	−	−	−	−	nt	−	−	−	−	−	−	−	−	−	−	−	nt
	Gd12	−	−	−	−	−	−	nt	−	−	−	−	−	−	−	−	−	−	−	nt
	Gd13	−	−	−	−	−	−	nt	−	−	−	−	−	−	−	−	−	−	−	nt
	Gd14	−	−	−	−	−	−	nt	−	−	−	−	−	−	−	−	−	−	−	nt
	Gd16	−	−	−	−	−	−	nt	−	−	−	−	−	−	−	−	−	−	−	nt
	Gd17	−	−	−	−	−	−	nt	−	−	−	−	−	−	−	−	−	−	−	nt
	Ccy1	−	−	−	−	−	−	nt	−	−	−	−	−	−	−	−	−	−	−	nt
	Ccy19	−	−	−	−	−	−	nt	−	−	−	−	−	−	−	−	−	−	−	nt
	Ccy46	−	−	−	−	−	−	nt	−	−	−	−	−	−	−	−	−	−	−	nt
	Ccy74	−	−	−	−	−	−	nt	−	−	−	−	−	−	−	−	−	−	−	nt
	Ccy121	−	−	−	−	−	−	nt	−	−	−	−	−	−	−	−	−	−	−	nt
	Ccy118	−	−	−	−	−	−	nt	−	−	−	−	−	−	−	−	−	−	−	nt
	Ccy110	−	−	−	−	−	−	nt	−	−	−	−	−	−	−	−	−	−	−	nt
	Ccy113	−	−	−	−	−	−	nt	−	−	−	−	−	−	−	−	−	−	−	nt
	Ccy281	−	−	−	−	−	−	nt	−	−	−	−	−	−	−	−	−	−	−	nt
	Ccyn2B	−	−	−	−	−	−	nt	−	−	−	−	−	−	−	−	−	−	−	nt

Capsular typing was determined by PCR using the oligonucleotides given in Supplementary Table S2. The results were interpreted as described previously^14^: strains positive for PCR A, PCR A and PCR B or PCR A and PCR C were typed as A, strains positive for only PCR B were typed as B, and strains positive for only PCR C as C. Western blot analysis on polysaccharidic structures was performed using specific polyclonal rabbit antisera. *nt* non-typable

## Discussion

In our previous work, we showed that *C. canimorsus* is endowed with capsular polysaccharides and that the majority of human-isolated strains exhibit capsule type A, B, or C, suggesting that these three capsular serovars are more virulent to humans^13,14^. In this study, we confirmed the prevalence of *C. canimorsus* capsular serovars A, B, and C in a larger collection of human-isolated strains. Among the 102 strains within the CCUG, 16S rDNA sequence analyses showed that 96 were *C. canimorsus sensu stricto*, while 6 belonged to the closely related species *C. canis*; the misclassifications were due to the recognition of *C. canis* as a distinct species 26 years after the valid publication of *C. canimorsus*^2,3^. Serotyping the 96 *C. canimorsus* strains confirmed the prevalence of capsular serovars A, B, and C in strains isolated from human infections, with 86 of 96 strains (89.6%) belonging to these three serovars, with approximately 30% of each serovar. No significant difference was found in the prevalence of the A, B, and C serovars (Fisher’s exact test, with significance levels of *p* < 0.05, *p* = 0.2441 for A, *p* = 1 for B, and *p* = 0.589 for C) between the human-isolated *C. canimorsus* of the CCUG collection and the previously studied collection of strains isolated worldwide^14^. These data also confirmed that there are significantly more strains belonging to serovars A, B, and C among human isolates than among dog isolates (7.6%), confirming that these three serovars are more virulent than any of the others (Fig. 2c, d). In addition, our study confirms that the A, B, and C serovars are not restricted to one geographical area. In addition to the serovar A, B, and C strains, the CCUG collection contained seven (7.3%) *C. canimorsus* strains of serovar D. These data are in agreement with the finding that the D serovar is the fourth most prevalent serovar among *C. canimorsus* human isolates^14^. Thus, although they represent only 7–8% (ref. ^14^ and this study) of the human clinical isolates, serovar D strains should be considered to be virulent and considered for prophylaxis. In contrast to our previous findings^14^, we could not detect any serovar E strain among the CCUG collection, although we discovered two new capsular serovars, L and M, each with a limited (1%) prevalence in human infections. We also found one non-capsulated strain among the CCUG collection. Unfortunately, due to the lack of clinical data regarding the patients from whom all CCUG strains were isolated, at this point, we cannot draw any conclusions regarding correlation of the severity of the disease with the capsular serovars. Nevertheless, the present study confirms that serovars other than A–D may cause human infection but with a very low frequency. These serovars may be less virulent and infectious only for immunocompromised patients, although we have no data to test this hypothesis here. We also tested the two new L and M antisera against the collection of strains isolated from Swiss and Belgian dogs^3^ and found two Swiss *C. canimorsus* strains endowed with an L-type capsule. Thus, as we have shown before^14^, the serovars are internationally distributed. With 11 *C. canimorsus* serovars (A–M) characterized to date, we can type only 38.5% of the dog-isolated strains of our collection (20 of 52), thus reinforcing a hypothesis of the existence of a large repertoire of capsular serovars in dog-hosted *C. canimorsus*.

*C. canis* was originally isolated from healthy dogs and presumed to be a non-pathogenic species because it was found exclusively in dogs and not among the strains of *Capnocytophaga* spp. isolated from severe human infections. In addition, *C. canis* strains are missing several factors that are suspected to contribute to *C. canimorsus* pathogenicity, such as the ability to acquire iron from transferrin and proliferate in human serum, as well as the capacity to deglycosylate host glycoproteins and a lack of oxidase activity^3^. Since the description of the *C. canis* species in 2015^3^, only a few cases of human infection from *C. canis* have been reported in the literature. However, a *C. canis* strain was isolated from a cat wound, and another *C. canis* was recently reported to cause a human septicemia^4,5^. It was, thus, not totally unexpected to find some *C. canis* among the CCUG collection of clinical strains, although their limited number (6) compared to that of *C. canimorsus* (96) indicates that *C. canis* is much less involved in human infections than is *C. canimorsus*.

The *C. canis* strains previously isolated from human infections were both oxidase-positive^4,5^, differing from the majority of *C. canis* strains found in dog mouths^3^.

Among the six *C. canis* strains of the CCUG collection, we found two that were oxidase-positive, supporting the hypothesis that oxidase-positive *C. canis* strains are more virulent to humans than strains lacking this activity. We found one *C. canis* strain to be endowed with a serovar B capsule. This observation is of great interest, because it shows for the first time that this capsular serovar is not restricted to the *C. canimorsus* species. In addition, we identified four dog-isolated *C. canis* strains belonging to capsular serovar A and one isolate belonging to the F serovar, showing that several *C. canimorsus* serovars are present in the closely related *C. canis* species. We found 11.7% of dog-isolated *C. canis* belonging to the A serovar, indicating that this serovar is even more frequent in *C. canis* than in dog-isolated *C. canimorsus* (1.9%). In contrast, among the six human-isolated *C. canis* strains, we could not find any serovar A strain. Considering the limited number of clinical *C. canis* strains analyzed, we cannot draw any definitive conclusion, but we can hypothesize that, in *C. canis*, capsular serovar A strains are not more virulent than other serovars, as is the case for *C. canimorsus*. The same conclusion cannot be drawn for serovar B *C. canis*, which we found in one clinical isolate but not in dog-isolated *C. canis*. Most *C. canis* strains could not be typed with the 11 sera we generated, suggesting that, as in *C. canimorsus*, there is a large reservoir of capsular serovars in *C. canis*. In line with this hypothesis, we note *C. canis* strains G36 and G77, which are positive for PCR ABC but do not belong to the A, B, and C serovars. Their genomes likely encode the *wfdR* glycosyl transferase detected by the ABC PCR but not one or more genes that determine the A, B, or C capsular serovars.

Whether some *C. canis* serovars might be more virulent to humans remains unknown and will require further investigation.

The finding of *C. canis* strains having similar capsules as *C. canimorsus* raised the question as to whether the other closely related dog-hosted species, *C. cynodegmi*, might also have capsules with similar epitopes. Interestingly, the typing of 10 human-isolated *C. cynodegmi* strains revealed the presence of three serovar B strains, indicating that this capsular serovar is not restricted to the *C. canimorsus* and *C. canis* species. Among the dog-isolated *C. cynodegmi* strains, none could be typed with our 11 sera. The finding of 3 of 10 (30%) *C. cynodegmi* strains of serovar B among clinical isolates and none among dog isolates suggests that *C. cynodegmi* endowed with a B capsule is more virulent to humans (Fisher’s exact test with a significance level of *p* < 0.05, *p* = 0.0462). As stated before, these conclusions should be taken with caution, considering the limited number (26) of *C. cynodegmi* strains analyzed in this study.

In conclusion, our study confirms that *C. canimorsus* strains belonging to serovars A, B, and C are more virulent to humans than other strains. Since these serovars represent a low percentage of dog-isolated strains, we can infer that few dogs carry these dangerous strains. This conclusion is of great importance, because it contributes to an explanation for the paradox of the low incidence of the disease in spite of the abundance of dogs carrying *C. canimorsus*. This conclusion is important for the prevention of the disease, because the PCR test we have developed could be adapted to identify potentially more dangerous dogs, keeping in mind, however, that some *C. canis* strains do harbor an A capsule and some *C. cynodegmi* strains harbor a B capsule. A real-time PCR test could be developed, for example, to detect virulent *Capnocytophaga* strains directly in dog saliva samples. A step forward would then be to eradicate the strains from these animals, leading to a significant reduction in pathogen shedding and, finally, in human infections. Furthermore, we show, for the first time, that several capsular serovars (A, B, and F) are shared by the three dog-hosted *Capnocytophaga* species. The commonality of some capsular serovars in these three species might result from genetic transfers of the capsular genes. This hypothesis is supported by the genetic organization of the *C. canimorsus* capsular genes, which are organized in loci including genes coding for transposases^13^. Whether *Capnocytophaga* species exchange their capsular genes is an open question that requires further investigation.

## Materials and methods

### Bacterial strains and growth conditions

The *C. canimorsus*, *C. canis*, and *C. cynodegmi* bacterial strains used in this study are listed in Supplementary Table S1. For serotyping, bacteria were grown on heart infusion agar (BD Difco, Franklin Lakes, NJ, USA) plates supplemented with 5% sheep blood (Oxoid, Basingstoke, UK) and 20 µg/ml gentamicin (Sigma-Aldrich, Darmstadt, Germany) and incubated for 48 h at 37 °C with 5% CO_2_.

### Species identification by 16S rDNA sequencing

For 16S rRNA gene (16S rDNA) amplification by PCR, a single colony was resuspended in 100 µl ddH_2_O and heated for 10 min at 98 °C. One microliter was used as template for amplification of a 1.07 kb fragment of the 16S rDNA. The primers 27F and 1100R (Supplementary Table S2) were used at 0.4 mM concentration with 200 mM dNTP and 1 U Taq polymerase (NEB, Ipswich, MD, USA). PCR was carried out for five initial cycles (94 °C for 30 s, 60 °C for 2 min, 72 °C for 3 min), in which the annealing temperature was reduced by 1.5 °C/cycle, followed by 30 cycles (94 °C for 30 s, 52 °C for 90 s, 72 °C for 3 min) and a final elongation for 10 min at 72 °C. The 1.07 kb PCR product was extracted from a 1.2% agarose gel by NucleoSpin (Macherey Nagel, Düren, Germany). The cleaned PCR products were sequenced by Eurofins Genomics with the primers 27F, 685R, and 1100R (ref. ^15^ and ref. ^16^) (Supplementary Table S2). The consensus sequences were obtained using BioEdit software and then used to build a 16S phylogenetic tree using the Ribosomal Database Project Tree Builder tool (http://rdp.cme.msu.edu/).

### Antiserum production and adsorption

Anti-A to anti-I antiserum production is described in ref. ^14^. Rabbit polyclonal sera anti-L (strain G06) and anti-M (strain G58) and anti-G45 strain were prepared likewise according to ref. ^14^. Immunizations were performed at the Centre d’Economie Rurale (CER Groupe, Aye, Belgium). The CER animal welfare committee approved the animal handling and procedures. Sera were then adsorbed with a mix of 25 strains isolated from patients (Cc1 to Cc25, Supplementary Table S1) to obtain polyclonal antibodies specifically recognizing the capsular serovars against which the sera were raised. Adsorptions were performed by incubating 250 µl of antiserum with 6 × 10^9^ PFA-fixed bacteria on a rotating wheel at room temperature for at least 2 h. Bacteria were removed by successive centrifugations. The incubation and following centrifugations were repeated four times.

### ELISA

Capsular typing of *Capnocytophaga* strains by PCR, Western blotting and ELISA was performed as described in ref. ^14^.

## Electronic supplementary material

Supplementary figures

Supplementary tables
