# Supplementary material for: Capsular serovars of virulent Capnocytophaga canimorsus are shared by the closely related species C. canis and C. cynodegmi
Source: Emerg Microbes Infect. 2018 Jul 4;7:124. doi: 10.1038/s41426-018-0126-x (PMC6030092; doi:10.1038/s41426-018-0126-x)
Supplement: Supplementary file 1 — Supplementary figures [file 41426_2018_126_MOESM1_ESM.docx]

**Supplementary figures**

**
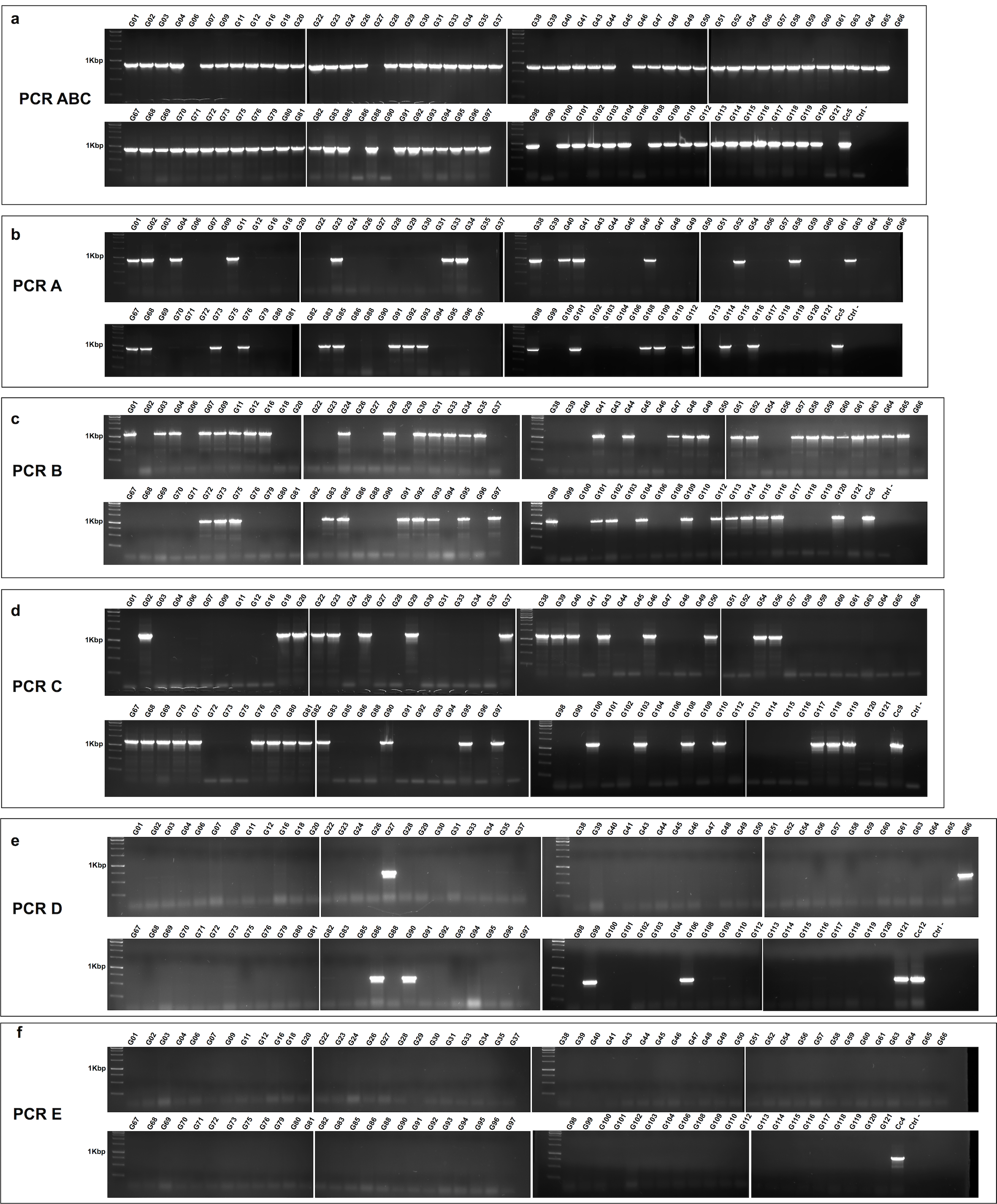
**

**Figure S1.** **Capsular typing by PCR of 96 human isolated *C. canimorsus* strains from the CCUG collection.**

Detection of capsular serovars A to E was performed using the oligonucleotides given in Supplementary Table S2.


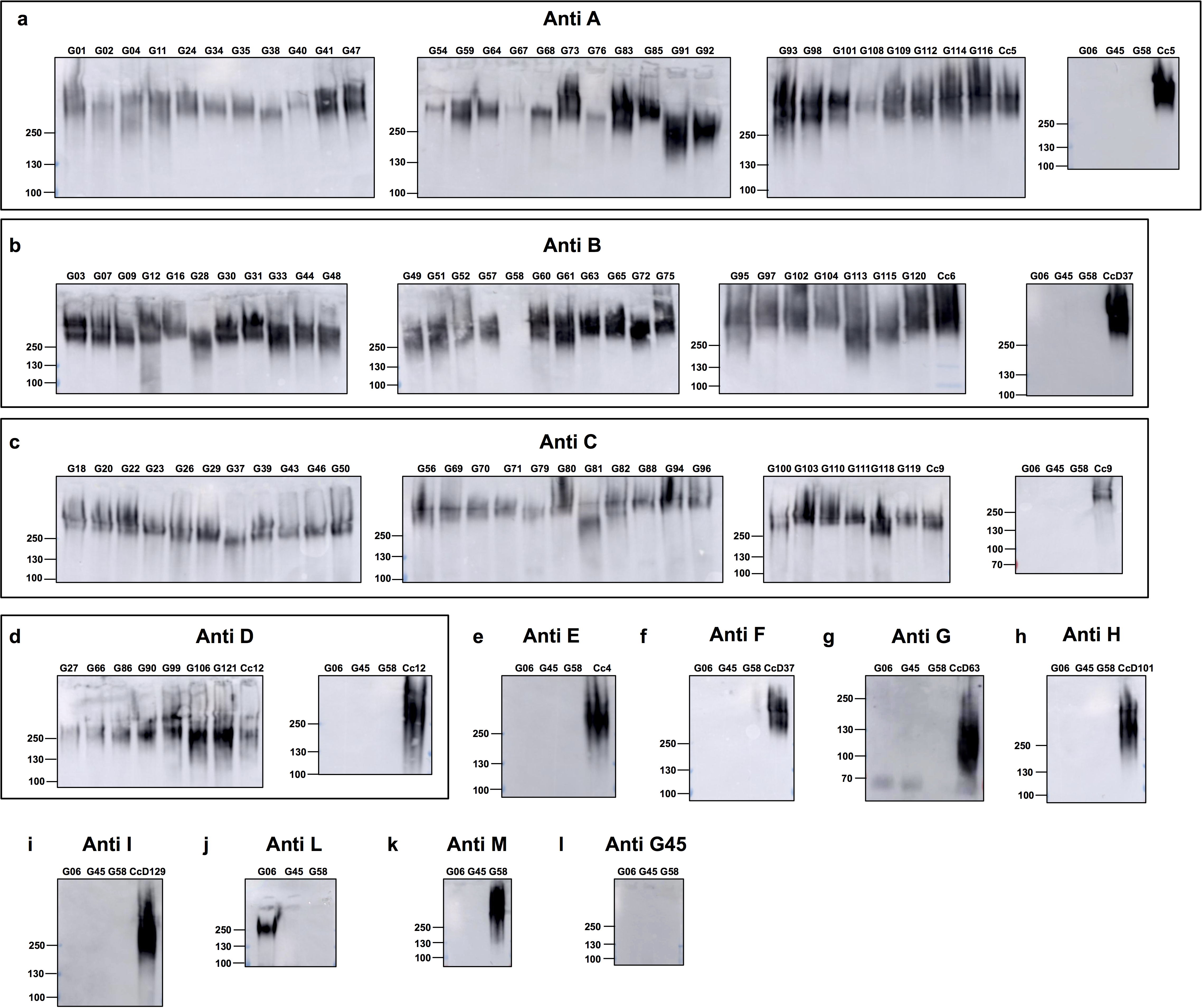


**Figure S2. Capsular typing by Western-blot of 96 human isolated *C. canimorsus* strains from the CCUG collection.**

Western blot analysis of proteinase K-treated lysates of *C. canimorsus* isolates was performed using the following sera: Y1C12-adsorbed anti-Cc5 (a), Cc6 Δ*wbuB*-adsorbed anti-Cc6 (b), Cc9 Δ*wbu*B-adsorbed anti-Cc9 (c), Cc12 Δ*wbtA*-adsorbed anti-Cc12 (d), anti-Cc4 adsorbed with human isolates Cc1-25 except Cc4 (e), anti CcD37 adsorbed with human isolates Cc1-25 (f), anti-CcD63 adsorbed with human isolates Cc1-25 (g), anti-CcD101 adsorbed with human isolates Cc1-25 (h), anti CcD129 adsorbed with human isolates Cc1-25 (i), anti-G06 adsorbed with human isolates Cc1-25 (j), anti-G58 adsorbed with human isolates Cc1-25 (k), anti-G45 (l). Numbers correspond to molecular weight markers in thousands.

**
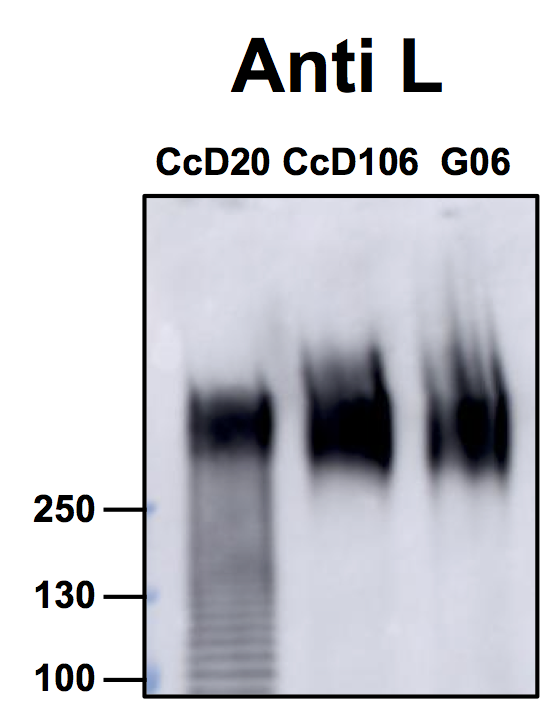
**

**Figure S3. Capsular serovar L detection by Western-blot in dog- isolated *C. canimorsus* strains.**

Western blot analysis of proteinase K-treated lysates of *C. canimorsus* isolates CcD20 and CcD106 was performed using anti-G06 adsorbed with human isolates Cc1-25 serum. Numbers correspond to molecular weight markers in thousands.


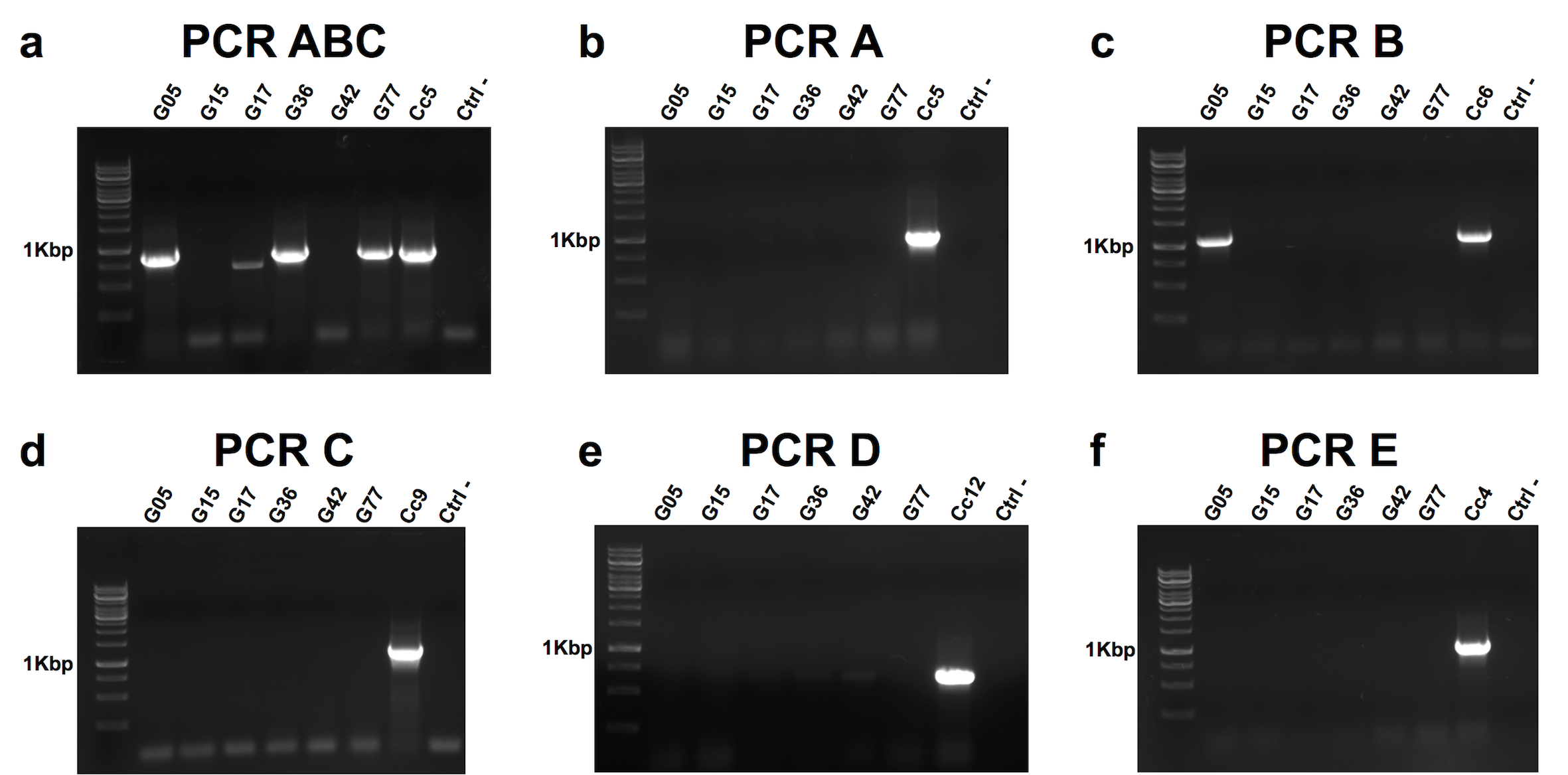


**Figure S4.** **Capsular typing by PCR of 6 human isolated *C. canis* strains from the CCUG collection.**

Detection of capsular serovars A to E was performed using the oligonucleotides given in Supplementary Table S2.


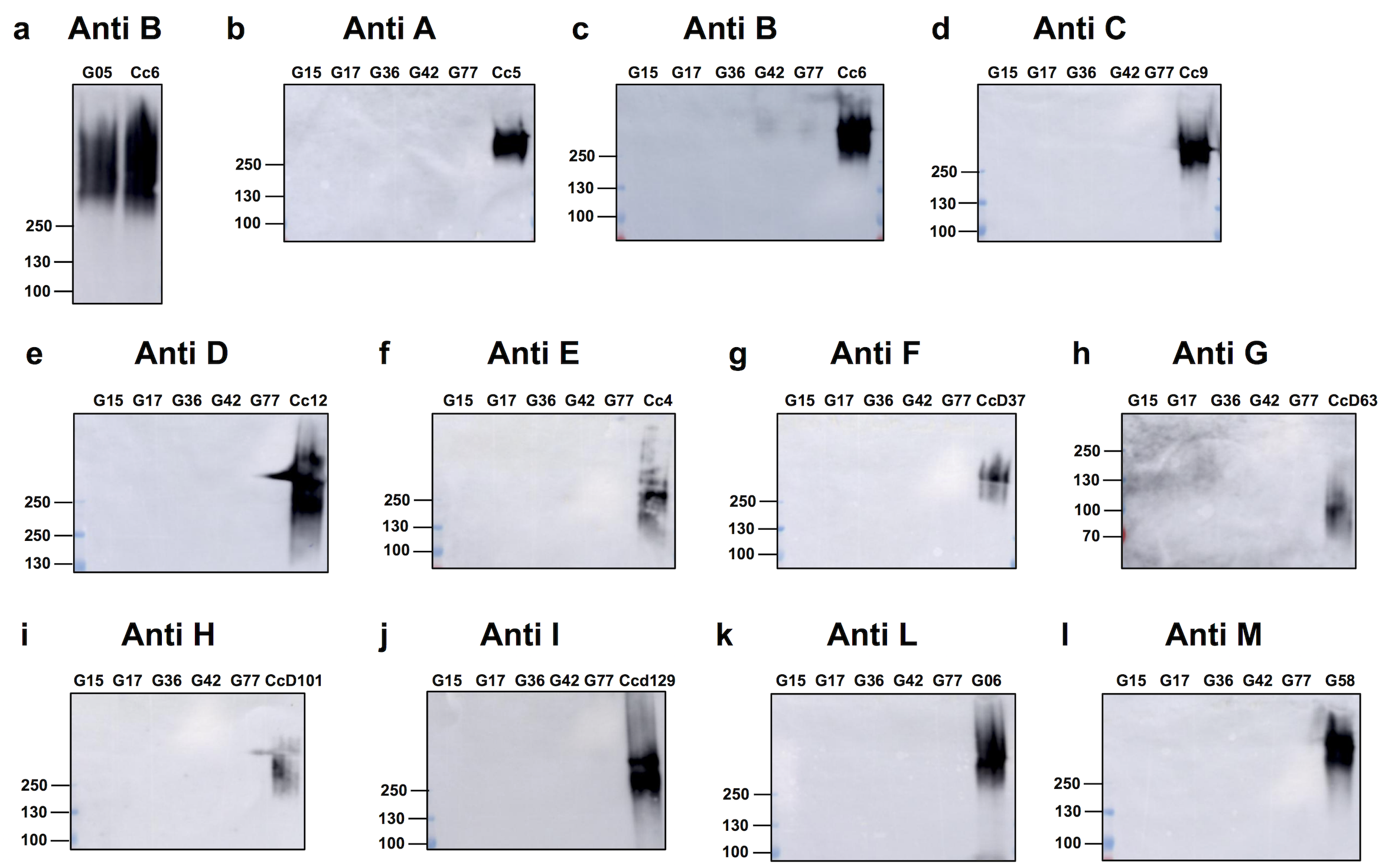


**Figure S5. Capsular typing by Western-blot of 6 human isolated *C. canis* strains from the CCUG collection.**

Western blot analysis of proteinase K-treated lysates of *C. canis* isolates was performed using the following sera: Cc6 Δ*wbuB*-adsorbed anti-Cc6 (a and c), Y1C12-adsorbed anti-Cc5 (b), Cc9 Δ*wbuB*-adsorbed anti-Cc9 (d), Cc12 Δ*wbtA*-adsorbed anti-Cc12 (e), anti-Cc4 adsorbed with human isolates Cc1-25 except Cc4 (f), anti-CcD37 adsorbed with human isolates Cc1-25 (g), anti-CcD63 adsorbed with human isolates Cc1-25 (h), anti-CcD101 adsorbed with human isolates Cc1-25 (i), anti-CcD129 adsorbed with human isolates Cc1-25 (j), anti-G06 adsorbed with human isolates Cc1-25 (k), anti-G58 adsorbed with human isolates Cc1-25 (l). Numbers correspond to molecular weight markers in thousands.

**
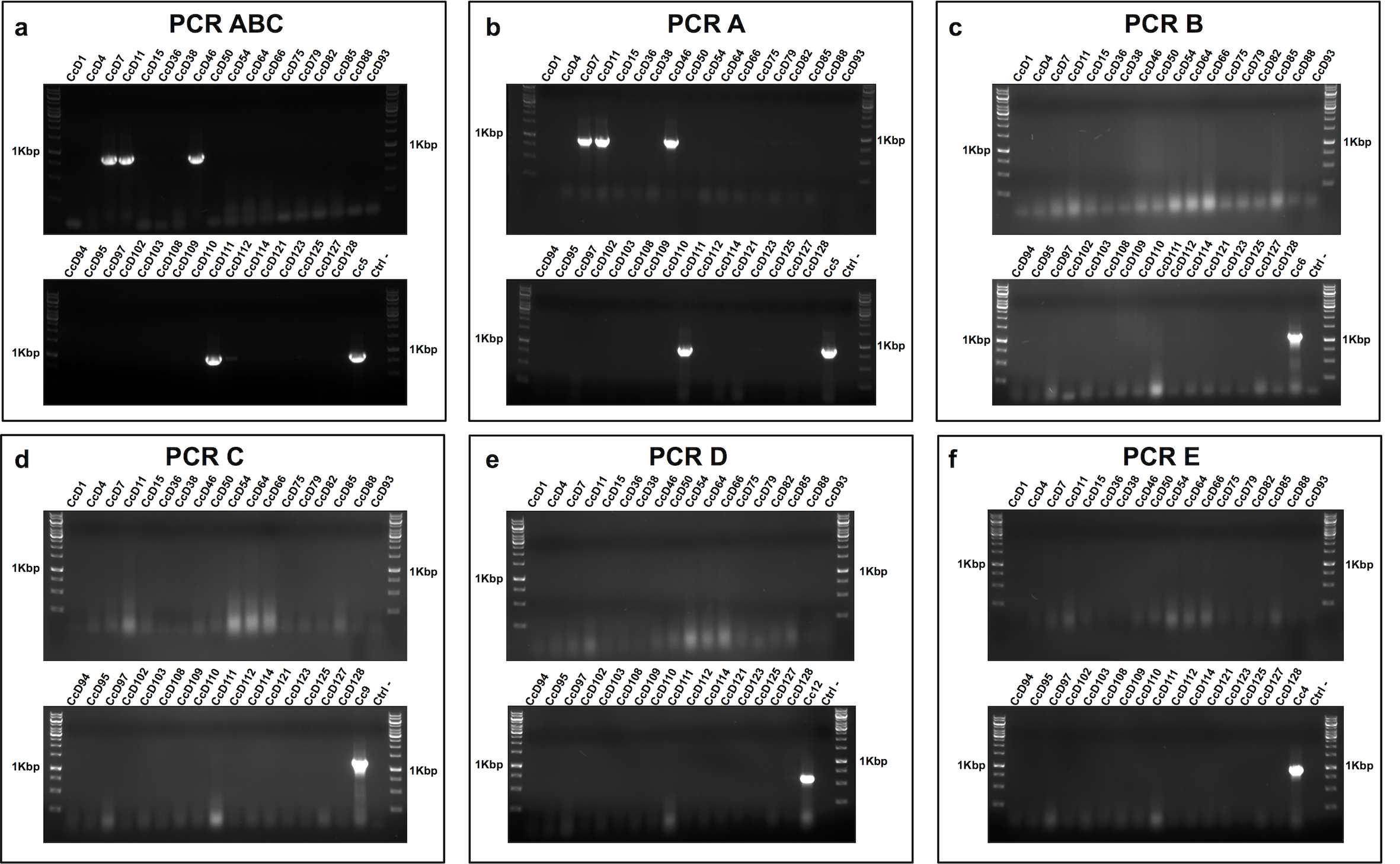
**

**Figure S6.** **Capsular typing by PCR of 34 dog-isolated *C. canis* strains.**

Detection of capsular serovars A to E was performed using the oligonucleotides given in Supplementary Table S2.


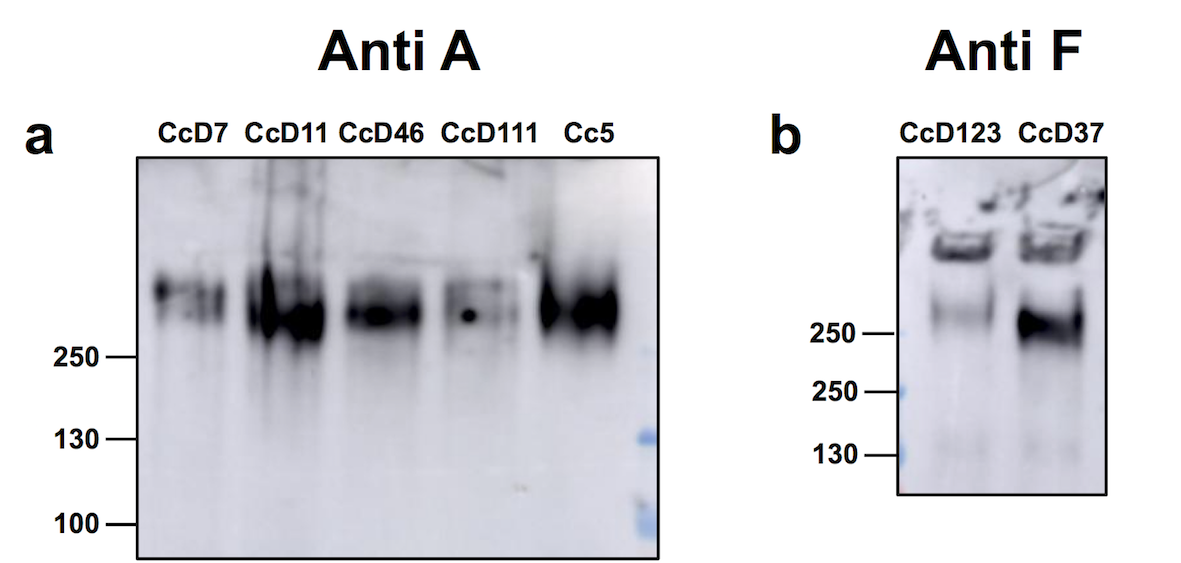


**Figure S7. Capsular serovar A and F detection by Western-blot in dog- isolated *C. canis* strains.**

Western blot analysis of proteinase K-treated lysates of *C. canis* isolates was performed using Y1C12-adsorbed anti-Cc5 (a) or anti-CcD63 adsorbed with human isolates Cc1-25 sera (b). Numbers correspond to molecular weight markers in thousands.

**
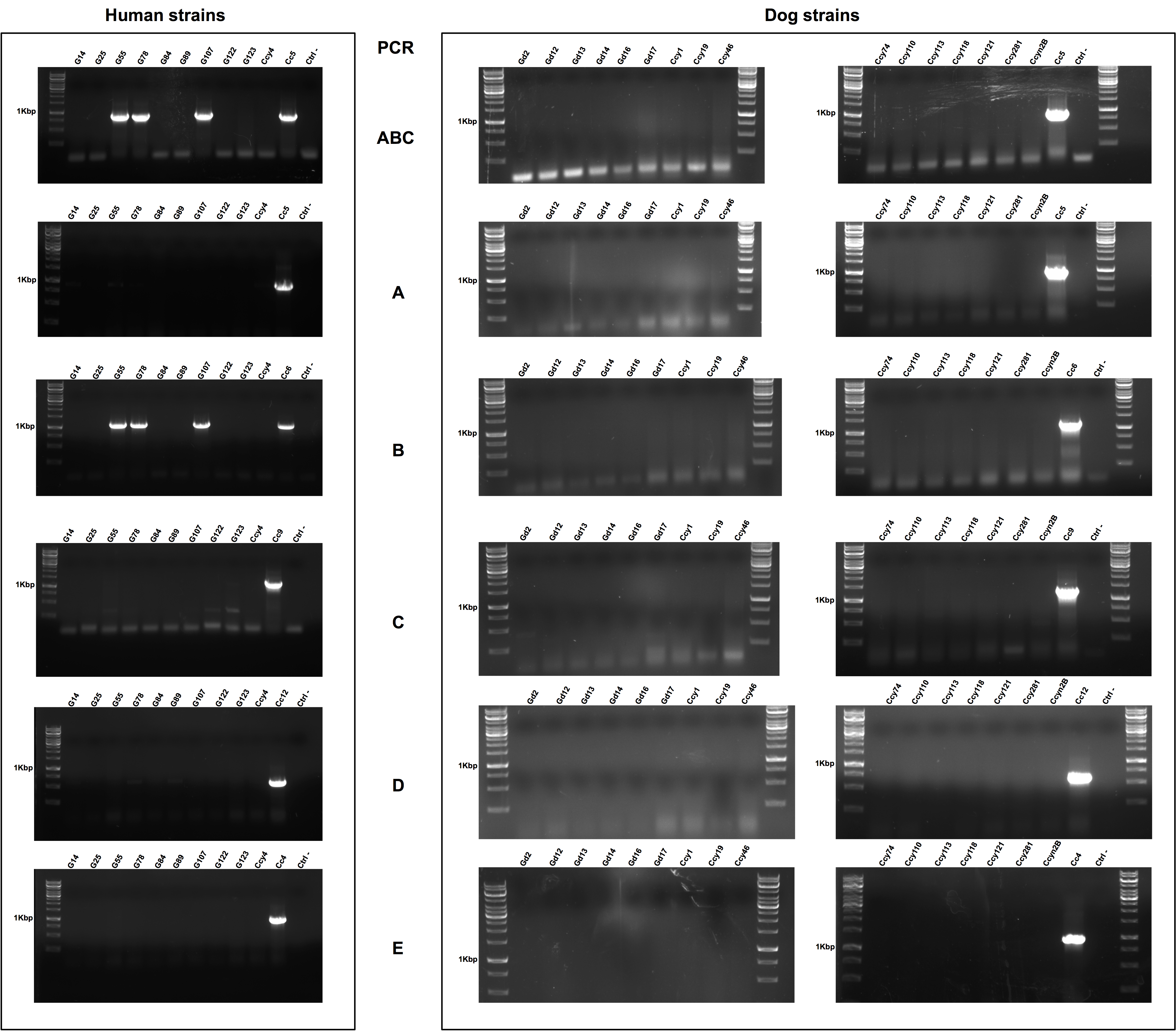
**

**Figure S8.** **Capsular typing by PCR of 10 human-isolated and 16 dog-isolated *C. cynodegmi* strains.**

Detection of capsular serovars A to E was performed using the oligonucleotides given in Supplementary Table S2.


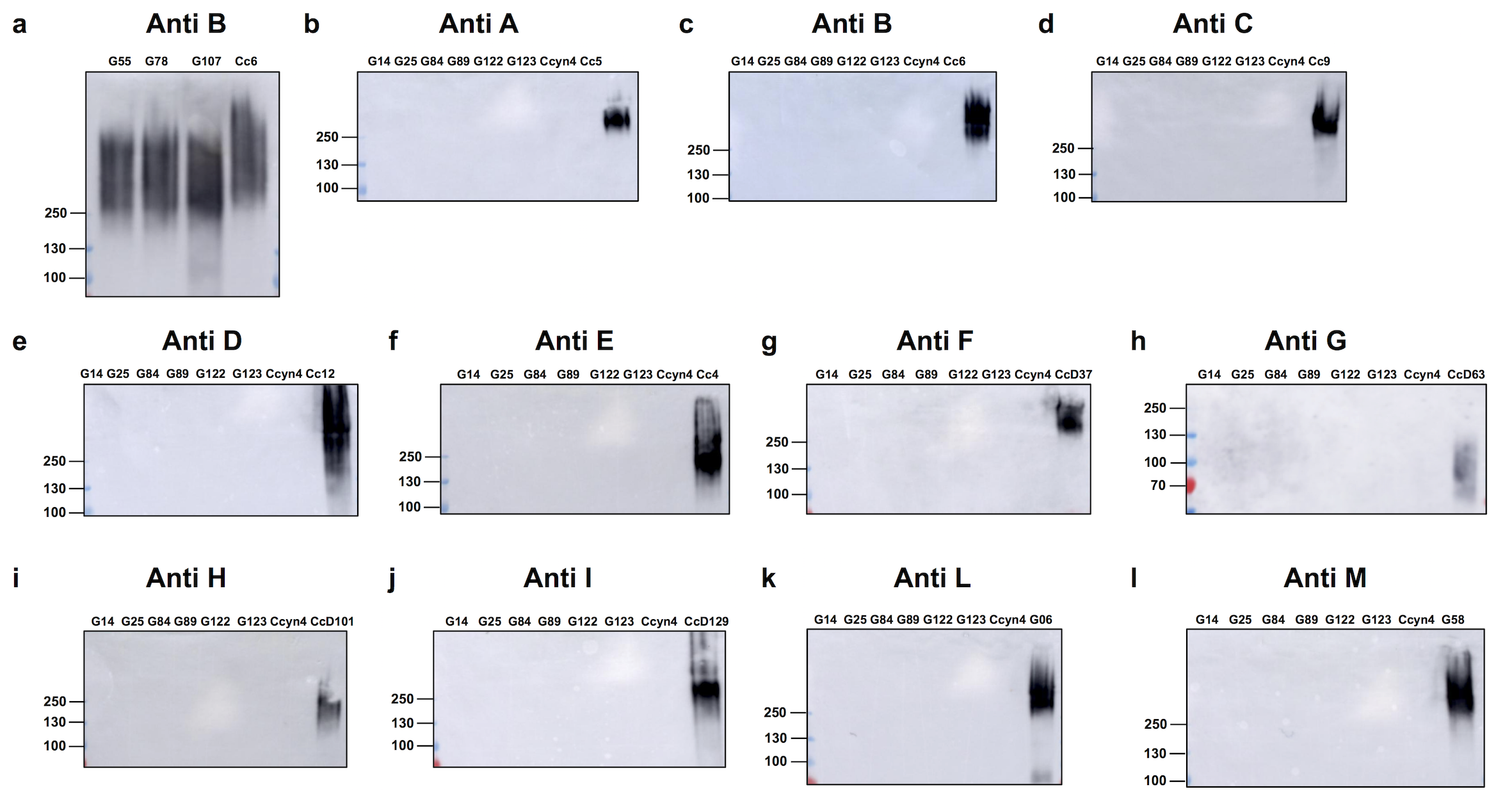


**Figure S9. Capsular typing by Western-blot of 10 human-isolated *C. cynodegmi* strains.**

Western blot analysis of proteinase K-treated lysates of *C. cynodegmi* isolates was performed using the following sera:, Cc6 Δ*wbuB*-adsorbed anti-Cc6 (a and c), Y1C12-adsorbed anti-Cc5 (b) Cc9 Δ*wbuB*-adsorbed anti-Cc9 (d), Cc12 Δ*wbtA*-adsorbed anti-Cc12 (e), anti-Cc4 adsorbed with human isolates Cc1-25 except Cc4 (f), anti-CcD37 adsorbed with human isolates Cc1-25 (g), anti-CcD63 adsorbed with human isolates Cc1-25 (h), anti-CcD101 adsorbed with human isolates Cc1-25 (i), anti-CcD129 adsorbed with human isolates Cc1-25 (j), anti-G06 adsorbed with human isolates Cc1-25 (k), anti-G58 adsorbed with human isolates Cc1-25 (l). Numbers correspond to molecular weight markers in thousands.


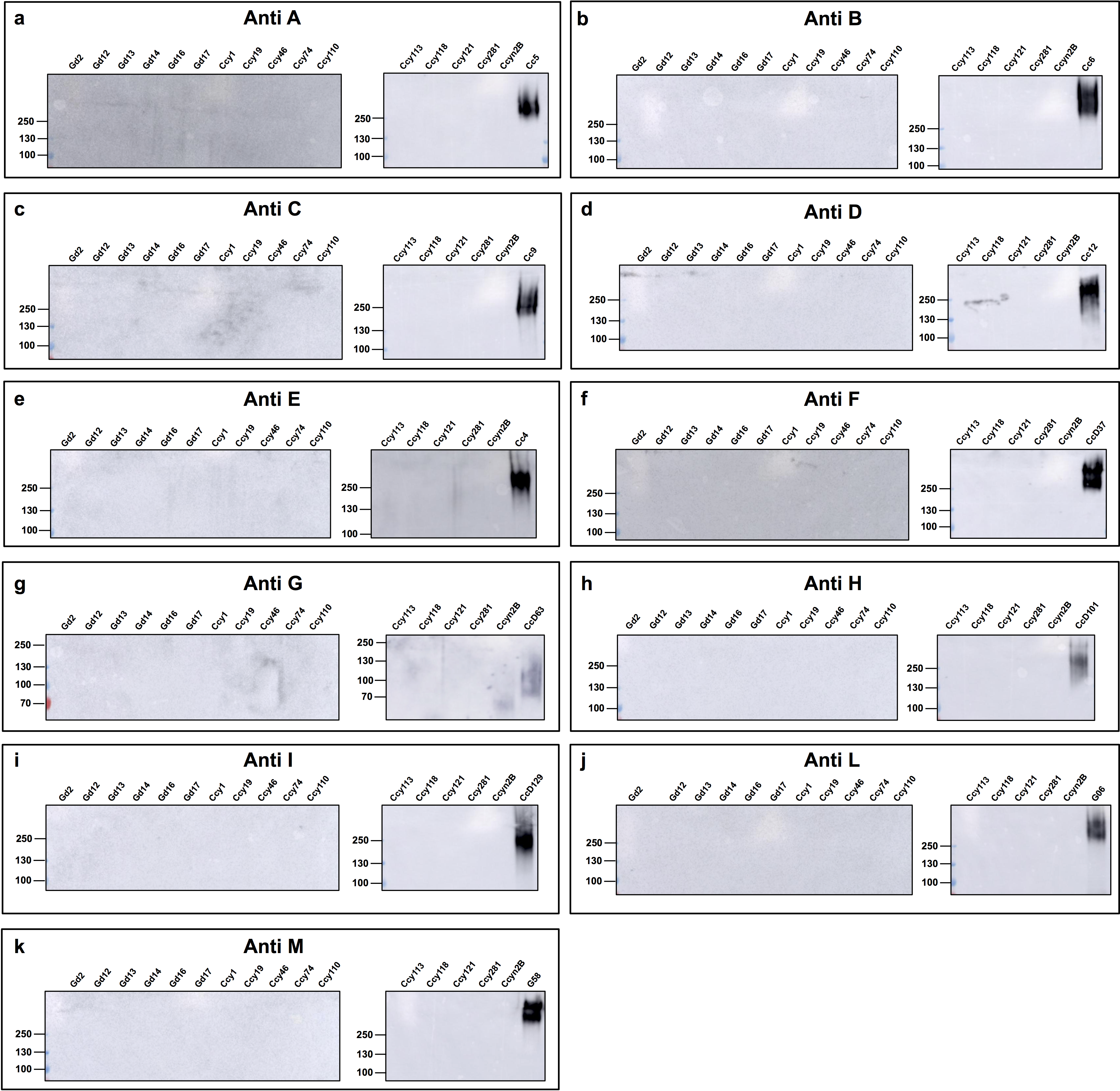


**Figure S10. Capsular typing by Western-blot of 16 dog-isolated *C. cynodegmi* strains.**

Western blot analysis of proteinase K-treated lysates of *C. cynodegmi* isolates was performed using the following sera: Y1C12-adsorbed anti-Cc5 (a), Cc6 Δ*wbuB*-adsorbed anti-Cc6 (b), Cc9 Δ*wbuB*-adsorbed anti-Cc9 (c), Cc12 Δ*wbtA*-adsorbed anti-Cc12 (d), anti-Cc4 adsorbed with human isolates Cc1-25 except Cc4 (e), anti-CcD37 adsorbed with human isolates Cc1-25 (f), anti-CcD63 adsorbed with human isolates Cc1-25 (g), anti-CcD101 adsorbed with human isolates Cc1-25 (h), anti-CcD129 adsorbed with human isolates Cc1-25 (i), anti-G06 adsorbed with human isolates Cc1-25 (j), anti-G58 adsorbed with human isolates Cc1-25 (k). Numbers correspond to molecular weight markers in thousands.
