# Supplementary material for: Capsular serovars of virulent Capnocytophaga canimorsus are shared by the closely related species C. canis and C. cynodegmi
Source: Emerg Microbes Infect. 2018 Jul 4;7:124. doi: 10.1038/s41426-018-0126-x (PMC6030092; doi:10.1038/s41426-018-0126-x)
Supplement: Supplementary file 2 — Supplementary tables [file 41426_2018_126_MOESM2_ESM.docx]

**Table S1. Strains of *Capnocytophaga* spp. used in this study**

| **Strains isolated from human** | | | | | |
| --- | --- | --- | --- | --- | --- |
| **Species** | **Strain** | **Collection accession numbers** | **Biological origin** | **Year of isolation** | **History and Geographical origin** |
| *C. canimorsus* | Cc1 | BCCM/LMG 11511; CCUG 17234;  SSI P810;  strain P810; | blood; after contact with dog (no dog bite) | 1981 | BCCM/LMG < CCUG SE < W.Frederiksen, SSI Copenhagen DK < J. Ursing, Univ. Malmö, Malmö SE |
| *C. canimorsus* | Cc2 | UNamur;  CCUG 70775 | blood, septicemia | 1989 | G.Wauters & M.Delmee, Clin. Univ. St Luc, Brussels BE |
| *C. canimorsus* | Cc3 | UNamur | blood, septicemia | 1990 | G.Wauters & M.Delmee < Sint-Jan Hospital, Brugge BE |
| *C. canimorsus* | Cc4 | UNamur;  CCUG 70776 | Prosthetic aortitis | 2010 | J.Schrenzel, Hopitaux Universitaires de Geneve, Geneva CH |
| *C. canimorsus* | Cc5 | BCCM/LMG 28512; CCUG 70777 | blood, fatal septicemia | 1995 | G.Wauters & M.Delmee < Clinic of Libramont, Libramont BE |
| *C. canimorsus* | Cc6 | UNamur;  CCUG 70778 | blood, septicemia | 1996 | KUL, Leuven BE |
| *C. canimorsus* | Cc7 | UNamur | blood, septicemia | 1998 | G.Wauters & M.Delmee. < KUL, Leuven BE |
| *C. canimorsus* | Cc8 | UNamur | blood, septicemia | 2004 | M.Delmee < Liege BE |
| *C. canimorsus* | Cc9 | BCCM/LMG 11510; CCUG 12569;  CDC A3626 | blood, septicemia | 1965 | BCCM/LMG < CCUG < E.Edling, Biodisk, Stockholm SE < R.Weaver, CDC Atlanta USA |
| *C. canimorsus* | Cc10 | BCCM/LMG 11541; CCUG 24741;  ATCC 35978;  CDC 8936;  CHD 32-1-74 | blood, septicemia;  after dog bite | 1989 | BCCM/LMG < MCCM < ATCC < R.Weaver, CDC, Atlanta USA < California Health Dept USA |
| *C. canimorsus* | Cc11 | BCCM/LMG 11551;  CCUG 70779;  MCCM 01373 | blood, septicemia | 1990 | BCCM/LM < MCCM < A.vonGraevenitz, Univ. Zurich CH |
| *C. canimorsus* | Cc12 | CCUG 53895T;  ATCC 35979T;  CDC 7120;  7120 | blood, septicemia;  after dog bite | 1961 | ATCC < R.Weaver. CDC Atlanta Georgia. California State Health Dept San Antonio Community Hospital, California USA |
| *C. canimorsus* | Cc13 | UNamur | blood, septicemia | 2008 | F.S.Stals, Laurentius Ziekenhuis, Roermond NL |
| *C. canimorsus* | Cc14 | UNamur | blood, septicemia | 2011 | R.Jarsumbeck, Medizinisches Labor Ostsachsen, Dresden DE |
| *C. canimorsus* | Cc15 | UNamur | blood, septicemia | 2011 | K.Mühlemann. University Hospital Bern. Switzerland |
| *C. canimorsus* | Cc16 | UNamur | blood, septicemia | … | G.Glupczynski. CHU Mont Godinne < D.Olivier. Hopital Univ. Erasme, Brussels. Belgium |
| *C. canimorsus* | Cc17 | UNamur | blood, septicemia | … | G. Glupczynski, CHU Mont Godinne < D. Olivier, Hopital Univ. Erasme. Brussels BE |
| *C. canimorsus* | Cc18 | UNamur | blood, septicemia | … | G.Glupczynski. CHU Mont Godinne < D.Olivier. Hopital Univ. Erasme, Brussels BE |
| *C. canimorsus* | Cc19 | UNamur | blood, septicemia | 2010 | A.Magnette. CHU Mont-Godinne < M.Delmée, < Clinique Saint Pierre. Ottignies BE |
| *C. canimorsus* | Cc20 | CCUG 55909 | blood, septicemia | 2008 | < E.Ek. Blood Dept. PHLS. Göteborg. Sweden < UK National External Quality assessment. Colindale. London UK |
| *C. canimorsus* | Cc21 | CCUG 60839 | blood, septicemia | 2011 | < E.Ek. Blood Dept. PHLS. Göteborg SE |
| *C. canimorsus* | Cc22 | CCUG 20318 | blood, septicemia | 1986 | < W.Frederiksen, SSI, Copenhagen DK |
| *C. canimorsus* | Cc23 | CCUG 48899 | blood, septicemia | 2004 | < V.Roux & D.Raoult, Marseille FR |
| *C. canimorsus* | Cc24 | CCUG 67384 | blood, septicemia; after dog bite | 2015 | < N.Kamenska, NÄL, Trollhätten SE |
| *C. canimorsus* | Cc25 | CCUG 66222 | blood, septicemia | 2014 | < I.Adlerberth, Blood Dept. PHLS. Sahlgrenska University Hospital, Göteborg SE |
| *C. canimorsus* | G01 | CCUG 15945 | blood; after dog bite | 1984 | < E.Törnqvist. PHL, Örebro SE |
| *C. canimorsus* | G02 | CCUG 16984 | blood; after dog bite | 1984 | < PHL, Uddevalla SE |
| *C. canimorsus* | G03 | CCUG 17235 | blood | 1983 | < W.Frederiksen, SSI, Copenhagen DK |
|  |  |  |  |  |  |
| *C. canimorsus* | G04 | CCUG 17236 | blood | 1982 | < W.Frederiksen, SSI, Copenhagen DK |
| *C. canimorsus* | G06 | CCUG 17817 | blood; after dog bite | 1985 | < H.Fritz, PHL Halmstad SE |
| *C. canimorsus* | G07 | CCUG 18811 | blood; after dog bite | 1986 | < Blood Dept, PHL, Göteborg SE |
| *C. canimorsus* | G09 | CCUG 19052 | blood | 1986 | < PHLS, Örebro SE |
| *C. canimorsus* | G11 | CCUG 19434 | blood | 1986 | < Blood Dept, PHL, Göteborg SE |
| *C. canimorsus* | G12 | CCUG 20314 | blood | 1984 | < W.Frederiksen, SSI, Copenhagen DK |
| *C. canimorsus* | G16 | CCUG 25188 | blood; after dog bite | 1989 | < PHLS, Kalmar SE |
| *C. canimorsus* | G18 | CCUG 28225 | blood, fatal septicemia; after dog bite | 1991 | < M.Rylander, Karolinska, PHL, Stockholm SE |
| *C. canimorsus* | G20 | CCUG 30491 | blood, sepsis; after dog bite | 1992 | < M.Ullberg, Karolinska PHL. Stockholm. SE |
| *C. canimorsus* | G22 | CCUG 31792 | blood, sepsis; after dog bite | 1993 | < B.Osterman, SMCL, Stockholm SE |
| *C. canimorsus* | G23 | CCUG 33174 | blood; after dog bite | 1994 | < J.Rydberg, Helsingborg SE |
| *C. canimorsus* | G24 | CCUG 33175 | blood; after dog bite | 1994 | < H.Fritz, PHL, Halmstad SE |
| *C. canimorsus* | G26 | CCUG 33783 | blood; after dog bite | 1994 | < J.Rydberg, Helsingborg SE |
| *C. canimorsus* | G27 | CCUG 34741 | blood | 1995 | < B.Claesson, PHL. Skövde. SE |
| *C. canimorsus* | G28 | CCUG 35893 | blood; after dog bite | 1996 | < E.Törnqvist. PHL, Örebro SE |
| *C. canimorsus* | G29 | CCUG 37293 | blood, sepsis; after animal bite | 1996 | < M.Thore, PHL, Västerås SE |
| *C. canimorsus* | G30 | CCUG 37801 | blood | 1997 | < PHL, Kristianstad SE |
| *C. canimorsus* | G31 | CCUG 38114 | blood | 1997 | < S.Bygdeman, PHLS, Huddinge SE |
| *C. canimorsus* | G33 | CCUG 38333 | blood; after dog bite | 1997 | < M.Granlund, PHL, Umeå SE |
| *C. canimorsus* | G34 | CCUG 38353A | blood | 1997 | < B.Carlsson, Kristianstad. SE |
| *C. canimorsus* | G35 | CCUG 38353B | blood | 1997 | < B.Carlsson. Kristianstad SE |
| *C. canimorsus* | G37 | CCUG 38694 | blood | 1997 | < Eastern Hospital, Göteborg SE |
| *C. canimorsus* | G38 | CCUG 20313 | blood | 1984 | < W.Frederiksen, SSI. Copenhagen DK |
| *C. canimorsus* | G39 | CCUG 20315 | blood | 1985 | < W.Frederiksen, SSI, Copenhagen DK |
| *C. canimorsus* | G40 | CCUG 20316 | blood | 1986 | < W.Frederiksen, SSI, Copenhagen DK |
| *C. canimorsus* | G41 | CCUG 20317 | blood | 1986 | < W.Frederiksen. SSI. Copenhagen. DK |
| *C. canimorsus* | G43 | CCUG 52283 | blood | 2005 | < K.Gullsby, Gävle SE |
| *C. canimorsus* | G44 | CCUG 53076 | blood; after dog bite | 2006 | < B.Olsson, Eastern.Hospital, Göteborg SE |
| *C. canimorsus* | G45 | CCUG 53302 | blood | 2006 | < K.Nilsson, PHLS, Falun SE |
| *C. canimorsus* | G46 | CCUG 53638 | blood; after dog bite | 2006 | < B.Björling, PHLS, Falun SE |
| *C. canimorsus* | G47 | CCUG 54209 | blood | 2006 | < C.Stenberg, Växjö SE |
| *C. canimorsus* | G48 | CCUG 55023 | blood, wound; contact with dog saliva | 2007 | < A.Pettersson, PHLS, Falun SE |
| *C. canimorsus* | G49 | CCUG 55116 | blood | 2007 | < O.Gustavsson, PHLS, Linköping SE |
| *C. canimorsus* | G50 | CCUG 55256A | blood | 2007 | < K.Dohse, Uppsala SE |
| *C. canimorsus* | G51 | CCUG 55264 | blood | 2007 | < C.Jendle, Karlstad SE |
| *C. canimorsus* | G52 | CCUG 55410 | blood, wound; contact with dog saliva | 2007 | < A.Nyberg, PHLS, Sundsvall SE |
| *C. canimorsus* | G54 | CCUG 57799 | blood; after dog bite | 2009 | < K.Wallgren, Gävle SE |
| *C. canimorsus* | G56 | CCUG 57915 | blood | 2009 | <E.Svensson. Blood Dept. PHLS, Göteborg SE |
| *C. canimorsus* | G57 | CCUG 58828 | cerebrospinal fluid | 2009 | < E. Halldin, Västeras SE |
| *C. canimorsus* | G58 | CCUG 61481 | blood | 2011 | < A.Andersson, Västeras SE |
| *C. canimorsus* | G59 | CCUG 61532 | blood | 2011 | < B.Jönsson, Blood Dept, Göteborg SE |
| *C. canimorsus* | G60 | CCUG 62838 | blood | 2012 | < Blood Dept. Göteborg SE |
| *C. canimorsus* | G61 | CCUG 64543 | blood | 2013 | < K.Gullsby, Gävle SE |
| *C. canimorsus* | G63 | CCUG 64791 | blood; after dog bite | 2013 | < M.Ygge, Sunderby sjh. Lulea SE |
| *C. canimorsus* | G64 | CCUG 65313 | blood | 2014 | < A.Svensson, Boras SE |
| *C. canimorsus* | G65 | CCUG 65314 | blood; after dog bite | 2014 | < L.Ask, Gen Diagn Dept. Göteborg SE < UNEQNAS |
| *C. canimorsus* | G66 | CCUG 65344 | blood | 2014 | <K.Wahlander, Linköping SE |
| *C. canimorsus* | G67 | CCUG 66143 | blood | 2014 | < N.Kamenska, NÄL, Trollhättan SE |
| *C. canimorsus* | G68 | CCUG 38828 | blood | 1997 | < B.Carlsson,.Kristianstad.SE |
| *C. canimorsus* | G69 | CCUG 38907 | blood; after dog bite | 1997 | < H.Gnarpe, PHL, Gävle SE |
| *C. canimorsus* | G70 | CCUG 38937 | blood; after dog bite | 1998 | < M.Ullberg. Karolinska PHL. Stockholm. SE |
| *C. canimorsus* | G71 | CCUG 38985 | blood; after dog bite | 1998 | < S.Bernander. KS.Stockholm. SE |
| *C. canimorsus* | G72 | CCUG 39024 | blood | 1998 | < PHL. Kalmar. SE |
| *C. canimorsus* | G73 | CCUG 39555 | blood | 1998 | < H.Gnarpe. PHL. Gävle. SE |
| *C. canimorsus* | G75 | CCUG 41543 | blood; after dog bite | 1998 | < E.Törnqvist. PHL. Örebro. SE |
| *C. canimorsus* | G76 | CCUG 42120 | blood; after dog bite | 1999 | < A.Nyberg. PHL. Sundsvall. SE |
| *C. canimorsus* | G79 | CCUG 42561 | blood | 1999 | < S.Johansson.PHLS.Halmstad.SE |
| *C. canimorsus* | G80 | CCUG 42590 | blood, sepsis | 1999 | < Blood Dept.. PHLS. Linköping. SE |
| *C. canimorsus* | G81 | CCUG 42656 | blood; after contact with dog | 1999 | < B.Carlsson. Kristianstad. SE |
| *C. canimorsus* | G82 | CCUG 42737 | blood | 1999 | < Blood Dept.. PHLS. Linköping. SE |
| *C. canimorsus* | G83 | CCUG 42749 | blood; after dog bite | 1999 | < PHL. Borås. SE |
| *C. canimorsus* | G85 | CCUG 43148 | blood | 1999 | < PHL. Kalmar. SE |
| *C. canimorsus* | G86 | CCUG 43240 | blood | 2000 | < Blood Dept..PHLS.Linköping.SE |
| *C. canimorsus* | G88 | CCUG 43393 | blood | 2000 | < I.Juhlander. PHLS. Huddinge. SE |
| *C. canimorsus* | G90 | CCUG 43421 | Human | 2000 | < PHLS. Sundsvall. SE |
| *C. canimorsus* | G91 | CCUG 43882 | blood; after dog bite | 2000 | < B.-M.Ellis. PHL. Karlskrona. SE |
| *C. canimorsus* | G92 | CCUG 44441 | blood | 2000 | < PHLS. Sundsvall. SE |
| *C. canimorsus* | G93 | CCUG 44797 | blood; after dog bite | 2001 | < Nova Medical. KSS. Skövde. SE |
| *C. canimorsus* | G94 | CCUG 44883 | blood | 2001 | < G.Kahlmeter. PHL. Växjö. SE |
| *C. canimorsus* | G95 | CCUG 45195 | blood | 2001 | < Nova Medical. KSS.S kövde. SE |
| *C. canimorsus* | G96 | CCUG 45528 | blood | 2001 | < PHL. Kalmar. SE |
| *C. canimorsus* | G97 | CCUG 45633 | blood | 2001 | < M.Sellin. PHLS. Umeå. SE |
| *C. canimorsus* | G98 | CCUG 45634 | blood; after dog bite | 2001 | < M.Sellin. PHLS. Umeå. SE |
| *C. canimorsus* | G99 | CCUG 46036 | blood; after dog bite | 2001 | < A.Wistedt. PHLS. Kalmar. SE |
| *C. canimorsus* | G100 | CCUG 46037 | blood | 2001 | < T.Ahlqvist. PHLS. Karlstad. SE |
| *C. canimorsus* | G101 | CCUG 46466 | blood; abdominal pain | 2002 | < T.Kjerstadius, PHL, Karlstad SE |
| *C. canimorsus* | G102 | CCUG 46648 | blood | 2002 | < A.Nyberg, PHLS, Sundsvall SE |
| *C. canimorsus* | G103 | CCUG 46752 | blood | 2002 | < T.Ahlqvist, PHLS, Karlstad SE |
| *C. canimorsus* | G104 | CCUG 46768 | blood | 2002 | < Å.Nordius, Mälarhospital, EskilstunaSE |
| *C. canimorsus* | G106 | CCUG 47403 | blood; after dog bite | 2003 | < S.Johansson, Blood Dept. PHLS, Göteborg SE < Colindale. London. UK |
| *C. canimorsus* | G108 | CCUG 48013 | blood; after dog bite | 2003 | < I.Esbjörnsson, PHLS, Borås SE |
| *C. canimorsus* | G109 | CCUG 48042 | blood; after dog bite | 2003 | < G.Öman, PHLS, Borås SE |
| *C. canimorsus* | G110 | CCUG 48606 | blood; after dog bite | 2003 | < Å.Nordius, Capio Diagn, Skövde SE |
| *C. canimorsus* | G112 | CCUG 49582 | blood | 2004 | < Mälarhospital, Eskilstuna SE |
| *C. canimorsus* | G113 | CCUG 49742 | blood | 2004 | < M.Andersson, PHLS, KML, Gävle SE |
| *C. canimorsus* | G114 | CCUG 50631 | blood | 2005 | < PHLS, Sundsvall SE |
| *C. canimorsus* | G115 | CCUG 50848 | blood | 2005 | < T.Ahlqvist, PHLS, Karlstad SE |
| *C. canimorsus* | G116 | CCUG 18951 A | blood; after dog bite | 1986 | < E.Berntsson, Blood Dept. PHLS, Göteborg SE |
| *C. canimorsus* | G117 | CCUG 30050 | blood; fever, pneumonia | 1992 | < G.Banck, PHL,Växjö SE |
| *C. canimorsus* | G118 | CCUG 38302 | blood; fever | 1997 | < B.Carlsson, Kristianstad SE |
| *C. canimorsus* | G119 | CCUG 71155 | blood; after dog bite | 2017 | < Blood Dept, Göteborg SE |
| *C. canimorsus* | G120 | CCUG 38709 | blood ; lymphoma | 1997 | < B.Carlsson, Kristianstad SE |
| *C. canimorsus* | G121 | CCUG 43025 | blood | 1999 | < Blood Dept., PHL, Göteborg SE < UKNEQAS. Collindale. UK |
| *C. canis* | G05 | CCUG 17663 | aorta fragment; endocarditis | 1985 | < Blood Dept., PHL, Göteborg SE |
| *C. canis* | G15 | CCUG 24824 | blood; after dog bite | 1989 | < B.Claesson, PHL, Skövde SE |
| *C. canis* | G17 | CCUG 25240 | blood; after dog bite | 1989 | < B.Selander, Lund SE |
| *C. canis* | G36 | CCUG 38354 | blood; after cat bite | 1997 | < B.Carlsson, Kristianstad SE |
| *C. canis* | G42 | CCUG 51338 | blood | 2005 | < T.Kjerstadius, PHL, Karlstad SE |
| *C. canis* | G77 | CCUG 42328 | blood | 1999 | < PHL, Borås SE |
| *C. cynodegmi* | G14 | CCUG 23144 | finger wound | 1988 | < PHL,Växjö SE |
| *C. cynodegmi* | G25 | CCUG 33224 | finger wound; after dog bite | 1994 | < Gen.Diagn.Dept., PHL, Göteborg SE |
| *C. cynodegmi* | G55 | CCUG 57842 | blood, meningitis; after dog bite | 2009 | < D.Heimer, PHLS, Västerås SE |
| *C. cynodegmi* | G78 | CCUG 42404 | blood; after contact with dog | 1999 | < PHLS, Sundsvall SE |
| *C. cynodegmi* | G84 | CCUG 42839 | wound; after dog bite | 1999 | < L.Bieber, PHLS, Växjö SE |
| *C. cynodegmi* | G89 | CCUG 43420 |  | 2000 | < PHLS. Sundsvall. Sweden |
| *C. cynodegmi* | G107 | CCUG 47845 | wound | 2003 | < A.Kötz, PHLS, Halmstad SE |
| *C. cynodegmi* | G122 | CCUG 30624;  LMG 11538 | hand wound | 1992 | < P.Vandamme, LMG, Gent BE < W.Mannheim, Marburg DE |
| *C. cynodegmi* | G123 | CCUG 38690 | bursitis, osteomyelitis | 1997 | < T.Kjerstadius, PHL, Karlstad SE |
| *C. cynodegmi* | Ccy4 | ATCC49045;  CDC 82010956 | hand wound | 1987 | < ATCC < R.Weaver < Missouri Div. Health < St. Francis Med. Ctr. Lab.. Cape Girardeau. MO. USA |
| **Strains isolated from dog** | | | | | |
| **Species** | **Strain** | **Collection** | **Biological origin** | **Year of isolation** | **History and Geographical origin** |
| *C. canimorsus* | CcD3 | UNamur | Dog mouth | 2008 | Switzerland |
| *C. canimorsus* | CcD5 | UNamur | Dog mouth | 2008 | Switzerland |
| *C. canimorsus* | CcD6 | UNamur | Dog mouth | 2008 | Switzerland |
| *C. canimorsus* | CcD10 | UNamur | Dog mouth | 2008 | Switzerland |
| *C. canimorsus* | CcD13 | UNamur | Dog mouth | 2008 | Switzerland |
| *C. canimorsus* | CcD16 | UNamur | Dog mouth | 2008 | Switzerland |
| *C. canimorsus* | CcD18 | UNamur | Dog mouth | 2008 | Switzerland |
| *C. canimorsus* | CcD20 | UNamur | Dog mouth | 2008 | Switzerland |
| *C. canimorsus* | CcD25 | UNamur | Dog mouth | 2008 | Switzerland |
| *C. canimorsus* | CcD33 | UNamur | Dog mouth | 2008 | Switzerland |
| *C. canimorsus* | CcD34 | UNamur | Dog mouth | 2008 | Switzerland |
| *C. canimorsus* | CcD35 | UNamur | Dog mouth | 2008 | Switzerland |
| *C. canimorsus* | CcD37 | UNamur | Dog mouth | 2008 | Switzerland |
| *C. canimorsus* | CcD39 | UNamur | Dog mouth | 2008 | Switzerland |
| *C. canimorsus* | CcD40 | UNamur | Dog mouth | 2008 | Switzerland |
| *C. canimorsus* | CcD43 | UNamur | Dog mouth | 2008 | Switzerland |
| *C. canimorsus* | CcD44 | UNamur | Dog mouth | 2008 | Switzerland |
| *C. canimorsus* | CcD47 | UNamur | Dog mouth | 2008 | Switzerland |
| *C. canimorsus* | CcD51 | UNamur | Dog mouth | 2008 | Switzerland |
| *C. canimorsus* | CcD52 | UNamur | Dog mouth | 2008 | Switzerland |
| *C. canimorsus* | CcD53 | UNamur | Dog mouth | 2008 | Switzerland |
| *C. canimorsus* | CcD57 | UNamur | Dog mouth | 2008 | Switzerland |
| *C. canimorsus* | CcD58 | UNamur | Dog mouth | 2008 | Switzerland |
| *C. canimorsus* | CcD63 | UNamur | Dog mouth | 2008 | Switzerland |
| *C. canimorsus* | CcD68 | UNamur | Dog mouth | 2008 | Switzerland |
| *C. canimorsus* | CcD69 | UNamur | Dog mouth | 2008 | Switzerland |
| *C. canimorsus* | CcD71 | UNamur | Dog mouth | 2008 | Switzerland |
| *C. canimorsus* | CcD73 | UNamur | Dog mouth | 2008 | Switzerland |
| *C. canimorsus* | CcD76 | UNamur | Dog mouth | 2008 | Switzerland |
| *C. canimorsus* | CcD77 | UNamur | Dog mouth | 2008 | Switzerland |
| *C. canimorsus* | CcD80 | UNamur | Dog mouth | 2008 | Switzerland |
| *C. canimorsus* | CcD81 | UNamur | Dog mouth | 2008 | Switzerland |
| *C. canimorsus* | CcD84 | UNamur | Dog mouth | 2008 | Switzerland |
| *C. canimorsus* | CcD89 | UNamur | Dog mouth | 2008 | Switzerland |
| *C. canimorsus* | CcD96 | UNamur | Dog mouth | 2008 | Switzerland |
| *C. canimorsus* | CcD101 | UNamur | Dog mouth | 2008 | Switzerland |
| *C. canimorsus* | CcD104 | UNamur | Dog mouth | 2005 | Switzerland |
| *C. canimorsus* | CcD105 | UNamur | Dog mouth | 2005 | Switzerland |
| *C. canimorsus* | CcD106 | UNamur | Dog mouth | 2006 | Switzerland |
| *C. canimorsus* | CcD113 | UNamur | Dog mouth | 2014 | Belgium |
| *C. canimorsus* | CcD115 | UNamur | Dog mouth | 2014 | Flawin. Belgium |
| *C. canimorsus* | CcD116 | UNamur | Dog mouth | 2014 | Hamoir. Belgium |
| *C. canimorsus* | CcD117 | UNamur | Dog mouth | 2014 | Belgium |
| *C. canimorsus* | CcD118 | UNamur | Dog mouth | 2014 | Wanze. Belgium |
| *C. canimorsus* | CcD119 | UNamur | Dog mouth | 2014 | Brussels. Belgium |
| *C. canimorsus* | CcD120 | UNamur | Dog mouth | 2014 | Belgium |
| *C. canimorsus* | CcD122 | UNamur | Dog mouth | 2014 | Belgium |
| *C. canimorsus* | Ccd124 | UNamur | Dog mouth | 2014 | Charleroi. Belgium |
| *C. canimorsus* | CcD126 | UNamur | Dog mouth | 2014 | Gerdinnes. Belgium |
| *C. canimorsus* | CcD129 | UNamur | Dog mouth | 2014 | Charleroi. Belgium |
| *C. canimorsus* | CcD130 | UNamur | Dog mouth | 2014 | Charleroi. Belgium |
| *C. canimorsus* | CcD131 | UNamur | Dog mouth | 2014 | Charleroi. Belgium |
| *C. canis* | CcD1 | UNamur | Dog mouth | 2008 | Switzerland |
| *C. canis* | CcD4 | UNamur | Dog mouth | 2008 | Switzerland |
| *C. canis* | CcD7 | UNamur | Dog mouth | 2008 | Switzerland |
| *C. canis* | CcD11 | UNamur | Dog mouth | 2008 | Switzerland |
| *C. canis* | CcD15 | UNamur | Dog mouth | 2008 | Switzerland |
| *C. canis* | CcD36 | UNamur | Dog mouth | 2008 | Switzerland |
| *C. canis* | CcD38 | UNamur | Dog mouth | 2008 | Switzerland |
| *C. canis* | CcD46 | UNamur | Dog mouth | 2008 | Switzerland |
| *C. canis* | CcD50 | UNamur | Dog mouth | 2008 | Switzerland |
| *C. canis* | CcD54 | UNamur | Dog mouth | 2008 | Switzerland |
| *C. canis* | CcD64 | UNamur | Dog mouth | 2008 | Switzerland |
| *C. canis* | CcD66 | UNamur | Dog mouth | 2008 | Switzerland |
| *C. canis* | CcD75 | UNamur | Dog mouth | 2008 | Switzerland |
| *C. canis* | CcD79 | UNamur | Dog mouth | 2008 | Switzerland |
| *C. canis* | CcD82 | UNamur | Dog mouth | 2008 | Switzerland |
| *C. canis* | CcD85 | UNamur | Dog mouth | 2008 | Switzerland |
| *C. canis* | CcD88 | UNamur | Dog mouth | 2008 | Switzerland |
| *C. canis* | CcD93 | UNamur | Dog mouth | 2008 | Switzerland |
| *C. canis* | CcD94 | UNamur | Dog mouth | 2008 | Switzerland |
| *C. canis* | CcD95 | UNamur | Dog mouth | 2008 | Switzerland |
| *C. canis* | CcD97 | UNamur | Dog mouth | 2008 | Switzerland |
| *C. canis* | CcD102 | UNamur | Dog mouth | 2008 | Switzerland |
| *C. canis* | CcD103 | UNamur | Dog mouth | 2008 | Switzerland |
| *C. canis* | CcD108 | UNamur | Dog mouth | 2011 | Sassari. Italy |
| *C. canis* | CcD109 | UNamur | Dog mouth | 2014 | Hamoir. Belgium |
| *C. canis* | CcD110 | UNamur | Dog mouth | 2014 | Hamoir. Belgium |
| *C. canis* | CcD111 | UNamur | Dog mouth | 2014 | Belgium |
| *C. canis* | CcD112 | UNamur | Dog mouth | 2014 | Belgium |
| *C. canis* | CcD114 | UNamur | Dog mouth | 2014 | Remouchamps. Belgium |
| *C. canis* | CcD121 | UNamur | Dog mouth | 2014 | Liege. Belgium |
| *C. canis* | CcD123 | UNamur | Dog mouth | 2014 | Belgium |
| *C. canis* | CcD125 | UNamur | Dog mouth | 2014 | Chatelet. Belgium |
| *C. canis* | CcD127 | UNamur | Dog mouth | 2014 | Idegem. Belgium |
| *C. canis* | CcD128 | UNamur | Dog mouth | 2014 | Gesves. Belgium |
| *C. cynodegmi* | Gd02 | CCUG 19141 | Dog mouth | 1986 | A.Pedersen, SVA, Uppsala SE < Örebro SE |
| *C. cynodegmi* | Gd12 | CCUG 30237 | Dog saliva | 1992 | L.Ullman, Växjö SE |
| *C. cynodegmi* | Gd13 | CCUG 30238 | Dog saliva | 1992 | L.Ullman, Växjö SE |
| *C. cynodegmi* | Gd14 | CCUG 19953 | Dog mouth | 1987 | H.Björk, SVA, Uppsala SE |
| *C. cynodegmi* | Gd16 | CCUG 20054 | Dog mouth | 1987 | H.Björk, SVA, Uppsala SE |
| *C. cynodegmi* | Gd17 | CCUG19954 | Dog pharynx | 1987 | H.Björk, SVA, Uppsala SE |
| *C. cynodegmi* | Ccy1 | ATCC 49044T;  CCUG 24742T;  MCCM 00247;  E6447 | Dog mouth | 1979 | ATCC < R Weaver, CDC, Atlanta USA < Virginia State Health Dept USA |
| *C. cynodegmi* | Ccy19 | UNamur | Dog mouth | 2008 | Switzerland |

**Table S2: Oligonucleotides used in this study**

| **Name** | **Sequence 5'-3'** | **Reference** |
| --- | --- | --- |
| 27F | AGAGTTTGATCCTGGCTCAG | ^15, 16^ |
| 1100R | GGGTTGCGCTCGTTG | ^15, 16^ |
| 685R | TCTACGCATTTCACCGCTAC | ^15, 16^ |
| SeroA-fw | CATACCATGGGAAAAAAAGTACCAATAGTTTTTATATTTAACC | ^14^ |
| SeroA-rev | CCGCTCGAGTCATTTTTTTATCTTTTTTAATATATTCCAC | ^14^ |
| SeroB-fw | CATACCATGGGAATTAACAAAATTCTAATAG | ^14^ |
| SeroB-rev | CCGCTCGAGTTATTTTTTATTTTCATTAG | ^14^ |
| SeroC-fw | GGCGTATATCGTTGCTATTTTGTATG | ^14^ |
| SeroC-rev | CTATTAATATTTTCATTGTACACCACTTC | ^14^ |
| SeroD-fw | GATTTAAAAAATATAGTATTTTAGGAATTATCG | ^14^ |
| SeroD-rev | CTATACTTGTTCCCACTTTTTAGTTTC | ^14^ |
| SeroE-fw | GGAGGAGGAAAAGTATTATTAGATTATC | ^14^ |
| SeroE-rev | CTATTCATAATTCTTAAAGATACTTATCAATTC | ^14^ |
| SeroABC-fw | CTTGGTTAGGTAAAGTTGCCTTAC | ^14^ |
| SeroABC-rev | CAACATTTCTCCCATCTTATAATCCC | ^14^ |

**Table S3. Prevalence of capsular serovars L and M in a collection of 52 strains of *C. canimorsus* isolated from dogs.**

Prevalence of capsular serovars L and M was determined by ELISA on heat-killed bacteria. The following sera were used: anti-G06 adsorbed with human isolates Cc1 to Cc25 (L); anti-G58 adsorbed with human isolates Cc1 to Cc25 (M). The reference trains for serovar L and M were used as negative controls. The readout of the ELISA was absorbance but results are expressed here as percentages of reactivity calculated with respect to the absorbance value obtained for the capsular reference strain. Values are expresses as means. Isolates with high reactivities are highlighted in grey.

| **Strain** | **Serovar L** | **Serovar M** |
| --- | --- | --- |
| CcD3 | 22.9 | 13.4 |
| CcD5 | 23.2 | 11.0 |
| CcD6 | 22.4 | 11.9 |
| CcD10 | 20.6 | 10.0 |
| CcD13 | 22.3 | 12.3 |
| CcD16 | 18.0 | 10.8 |
| CcD18 | 29.8 | 12.6 |
| CcD20 | 75.1 | 13.1 |
| CcD25 | 20.3 | 11.7 |
| CcD33 | 22.9 | 13.6 |
| CcD34 | 10.4 | 8.4 |
| CcD35 | 10.3 | 8.5 |
| CcD37 | 20.7 | 11.7 |
| CcD39 | 17.8 | 11.4 |
| CcD40 | 22.4 | 13.0 |
| CcD43 | 23.4 | 24.8 |
| CcD44 | 18.1 | 12.3 |
| CcD47 | 25.6 | 13.6 |
| CcD51 | 26.6 | 14.4 |
| CcD52 | 21.4 | 11.0 |
| CcD53 | 24.7 | 11.7 |
| CcD57 | 22.7 | 10.8 |
| CcD58 | 24.7 | 20.4 |
| CcD63 | 23.3 | 12.2 |
| CcD68 | 20.9 | 24.5 |
| CcD69 | 11.8 | 10.0 |
| CcD71 | 11.5 | 8.9 |
| CcD73 | 24.8 | 12.8 |
| CcD76 | 8.9 | 7.4 |
| CcD77 | 11.0 | 8.0 |
| CcD80 | 16.3 | 11.6 |
| CcD81 | 21.0 | 10.7 |
| CcD84 | 32.2 | 13.3 |
| CcD89 | 22.0 | 11.3 |
| CcD96 | 30.0 | 14.5 |
| CcD101 | 21.7 | 12.9 |
| CcD104 | 21.1 | 13.3 |
| CcD105 | 10.9 | 9.1 |
| CcD106 | 94.3 | 18.5 |
| CcD113 | 20.3 | 10.8 |
| CcD115 | 19.0 | 12.4 |
| CcD116 | 22.2 | 12.7 |
| CcD117 | 20.4 | 12.0 |
| CcD118 | 19.6 | 10.3 |
| CcD119 | 21.2 | 12.6 |
| CcD120 | 24.5 | 11.8 |
| CcD122 | 13.7 | 11.9 |
| Ccd124 | 22.0 | 11.6 |
| CcD126 | 16.4 | 13.9 |
| CcD129 | 22.9 | 13.7 |
| CcD130 | 24.9 | 24.4 |
| CcD131 | 18.4 | 12.5 |
| G06 | 100 | 13.4 |
| G58 | 31.0 | 100 |
